# A portable, nanopore-based genotyping platform for near real-time detection of *Puccinia graminis* f. sp. *tritici* lineages and fungicide sensitivity

**DOI:** 10.1186/s12864-025-11428-w

**Published:** 2025-04-01

**Authors:** Loizos Savva, Anthony Bryan, Dominik Vinopal, Oscar E. Gonzalez-Navarro, Zennah Kosgey, Kimani Cyrus Ndung’u, Jemal Tola Horo, Kitessa Gutu Danu, Messele Molla, Yoseph Alemayehu, David P. Hodson, Diane G.O. Saunders

**Affiliations:** 1 John Innes Centre, Norwich Research Park, Norwich, UK; 2 Kenya Agricultural and Livestock Research Organization (KALRO), Food Crops Research Centre, Njoro, Kenya; 3 Ambo Research Center, Ethiopian Institute of Agricultural Research (EIAR), Ambo, Ethiopia; 4 EIAR, National Agricultural Biotechnology Research Center, Holeta, Ethiopia; 5 International Maize and Wheat Improvement Center (CIMMYT), Addis Ababa, Ethiopia; 6 CIMMYT, Khumaltar, Lalitpur, Nepal

**Keywords:** *Puccinia graminis* f. sp. *tritici*, Wheat rusts, Disease diagnostics, Point-of-care, Disease surveillance, Nanopore sequencing, Fungicide resistance

## Abstract

**Background:**

Fungal plant disease outbreaks are increasing in both scale and frequency, posing severe threats to agroecosystem stability, native biodiversity and food security. Among these, the notorious wheat stem rust fungus, *Puccinia graminis* f.sp. *tritici* (*Pgt*), has threatened wheat production since the earliest days of agriculture. New *Pgt* strains continue to emerge and quickly spread over vast distances through the airborne dispersal of asexual urediniospores, triggering extensive disease outbreaks as these exotic *Pgt* strains often overcome resistance in dominant crop varieties of newly affected regions. This highlights the urgent need for a point-of-care, real-time *Pgt* genotyping platform to facilitate early detection of emerging *Pgt* strains.

**Results:**

In this study, we developed a simple amplicon-based re-sequencing platform for rapid genotyping of *Pgt* isolates. This system is built around a core set of 276 *Pgt* genes that we found are highly polymorphic between *Pgt* isolates and showed that the sequence of these genes alone could be used to accurately type *Pgt* strains to particular lineages. We also developed a simplistic DNA preparation method and an automated bioinformatic pipeline, to enable these *Pgt* gene markers to be sequenced and analysed rapidly using the MinION nanopore sequencing device. This approach successfully enabled the typing of *Pgt* strains within approximately 48 h of collecting *Pgt*-infected wheat samples, even in resource-limited locations in Kenya and Ethiopia. In addition, we incorporated monitoring capabilities for sequence variations in *Pgt* genes that encode targets of the azole and succinate dehydrogenase inhibitor fungicides, enabling real-time tracking of potential shifts in fungicide sensitivity.

**Conclusion:**

The newly developed *Pgt* Mobile And Real-time, PLant disEase (MARPLE) diagnostics platform we established, now allows precise typing of individual *Pgt* strains while simultaneously tracking changes in fungicide sensitivity, providing an early warning system for potential indicators of changes in the *Pgt* population and emerging fungicide resistance. Further integration of this *Pgt* MARPLE diagnostics platform into national surveillance programmes will support more informed management decisions and timely responses to *Pgt* disease outbreaks, helping reduce the devastating crop losses currently caused by this ‘cereal killer’.

**Supplementary Information:**

The online version contains supplementary material available at 10.1186/s12864-025-11428-w.

## Background

Fungal phytopathogens pose a significant threat to agroecosystem stability, native biodiversity and food security. In agriculture, every year fungal pathogens destroy 10–23% of all major calorie and commodity crops, with a further 10–20% lost to fungal infection post-harvest [1]. Rapid and accurate diagnostics of disease-causing fungal agents is essential to enact appropriate control measures and limit the scale of disease outbreaks. Traditionally, this was achieved primarily through the direct observation of macro- and microscopic fungal structures [2]. However, these approaches can lack resolution and sensitivity, leading to a greater emphasis in modern times on emerging molecular detection tools. Since the first DNA-based molecular detection strategies for fungal pathogens were introduced in the late 20th century, a wide array of methods has been developed to increase the reproducibility, sensitivity and specificity of fungal disease diagnostics [3]. This includes standard polymerase chain reaction (PCR), real-time quantitative PCR, restriction fragment length polymorphism (RFLP), amplified fragment length polymorphism (AFLP), denaturing-gradient gel electrophoresis (DGGE), and DNA-fingerprinting based on microsatellite and minisatellite DNA markers, among many others [4]. Due to their simplicity, PCR-based molecular markers remain widely employed in many laboratories for precise taxonomic classification and for analysing genetic variability between fungal strains. More recently, the demand for in-field instrumentation has driven the development of portable molecular diagnostics tools, such as loop-mediated isothermal amplification (LAMP), lateral flow assay (LFA) and recombinase polymerase amplification (RPA) [5]. These in-field applications have markedly accelerated the speed of molecular disease diagnostics.

Beyond species and strain definition, molecular diagnostic tools also play a crucial role in the rapid detection and monitoring of molecular mechanisms of antifungal resistance. In susceptible crop cultivars, fungicides act as the main line of defence to inhibit or eradicate the growth of harmful fungi. However, many classes of antifungal agents are used extensively across agricultural and clinical settings, leading to a global shift of fungal populations towards more resistant species and genera (e.g [6]). This includes azoles, which are the most widely used class of antifungal agents [7]. Azoles target the cytochrome 14-α demethylase (Cyp51) enzyme that is essential for ergosterol synthesis in fungi [8]. The most common resistance mechanism involves mutations in the *CYP51* coding region that can result in inadequate binding of azoles to the Cyp51 enzyme [9]. Another growing class of antifungal agents are the succinate dehydrogenase inhibitors (SDHIs), that act to inhibit mitochondrial respiration [10]. Similarly, resistance to SDHIs tends to occur via mutation, leading to amino acid substitutions in the SDHB, SDHC, and SDHD subunits of the succinate dehydrogenase complex targeted by SDHIs [10]. The rapid nature of these molecular detection techniques permits much earlier intervention, which is critical to reducing yield losses and the spread of fungal pathogens.

As the molecular disease detection toolbox continues to expand, emerging genomic-based techniques have started to radically transform the speed, resolution and reproducibility of fungal disease diagnostic strategies [11]. For instance, amplicon-based and shotgun metagenomic approaches have already proved fruitful for soilborne pathogens that are particularly problematic to diagnose, including the genera *Rhizoctonia* spp., *Fusarium* spp., *Verticillium* spp., which can cause 50–75% yield loss across many different crop species [12]. Genomic-based techniques are also starting to transition from the research arena into frontline point-of-care (PoC) infectious disease diagnostics, supported by exponential growth in publicly available genomic data to guide species and strain typing [11]. For instance, the portable, real-time MinION nanopore sequencer [13] is uniquely suited for PoC diagnostics, as first demonstrated for viruses using whole genome sequencing. Where deployment of the MinION sequencer helped in surveillance efforts for the Ebola virus in Guinea in 2014–2015 [14], Zika virus in Brazil in 2016 [15] and in diagnosing Cassava mosaic virus in sub-Saharan Africa in 2017–2018 [16], among others. Yet, applying similar whole-genome sequencing approaches directly to fungal pathogens, has proved challenging largely due to their vast genome sizes; typically, tens of thousands times larger than viruses [17]. This is further complicated by the obligate biotrophic lifestyle of many fungal plant pathogens, that are unable to be separated from their plant host that they depend on for survival [18]. To overcome these challenges, we previously devised a targeted re-sequencing approach that directly utilises infected plant material and focuses on PCR-amplification of a smaller subset of polymorphic pathogen genes for the wheat yellow (stripe) rust pathogen, *Puccinia striiformis* f. sp. *tritici*, *Pst*. When this smaller gene subset is sequenced on the MinION platform, these sequences alone are sufficient for typing individual genetic variants of *Pst* [19]. This new strategy, termed Mobile And Real-time PLant disEase (MARPLE) diagnostics, presents a framework for developing similar mobile, genomic-based, strain-level diagnostics strategies for other complex fungal pathogens.

In this study we focused on the causal agent of wheat stem rust (*Puccinia graminis* f. sp. *tritici* (*Pgt*)), which is the most damaging of the three wheat rust fungi [20]. We identified a core set of 276 *Pgt* genes that are highly polymorphic between *Pgt* strains and showed that the sequence of these genes alone could be used to accurately type *Pgt* strains to particular lineages. We also developed a simplistic DNA preparation method and automated bioinformatic pipeline to enable these *Pgt* gene markers to be sequenced using the MinION nanopore sequencing device directly in resource-limited locations. In addition, we built into this *Pgt* genotyping platform the ability to monitor sequence diversification in *Pgt* targets of the azole and SDHI fungicides. Thus, the *Pgt* MARPLE diagnostics platform we established can be used not only to accurately type individual *Pgt* strains, but also to simultaneously monitor for potential changes in fungicide sensitivity that can be used to help better guide management decisions and limit the impact of *Pgt* disease outbreaks.

## Methods

### Population genetic analysis of global *Pgt* isolates

A set of 165 *Pgt* genomic and transcriptomic datasets were gathered from public repositories (Additional file 1: Table S1 [21–26]). Each dataset was independently aligned to the *Pgt* reference genome (isolate CRL 75-36-700-3 [27]), using default settings and the Burrows-Wheeler Alignment (BWA), version 0.7.5 [28], or Star, version 2.5 [29] for genomic and transcriptomic datasets respectively. Following sequence alignment, each of the 165 *Pgt* transcriptomic datasets were trimmed at splice junctions using the *SplitNCigarReads* tool in the Genome Analysis Toolkit (GATK), version 4.0 [30]. Single nucleotide polymorphisms (SNPs) were identified using SAMtools version 0.1.19 [28] and those meeting a minimum depth of coverage (10x for genomic DNA and 20x for RNA-seq) were identified and used for downstream analysis as described previously [31]. Datasets were assessed to ensure a distribution of read counts for biallelic SNPs of 0.5 and at least 15% of gene sequences present using custom Python scripts [32]. The 86 datasets fulfilling these requirements were used for sequence alignments of the gene-coding regions of all genes with at least 60% breadth of coverage in at least 60% of the 86 samples (Additional file 1: Table S2). Phylogenetic trees were generated using an approximate maximum likelihood approach using FastTree version 2.1.9 [33] and visualised in iTol version 6 [34]. Subdivisions in the *Pgt* population were assessed by multivariate discriminant analysis of principal components (DAPC) implemented in the adegenet 2.1.1 package in the R environment [35]. A total of 19,250 biallelic SNP sites that introduced a synonymous change in at least one isolate were used in the analysis, with the optimum number of clusters determined using the Bayesian information criterion (BIC).

### Evaluation of *Pst* gene amplification

A detailed evaluation on the established *Pst* polymorphic gene set was conducted to determine characteristics that could inform *Pgt* marker gene selection. To this aim, a collection of datasets generated from 18 *Pst*-infected wheat samples that were produced using the established *Pst* MARPLE diagnostics methodology [19] were selected for analysis. This included 10 previously published datasets that were generated from amplification of the 242 highly polymorphic *Pst* genes [19] and 8 newly generated datasets resulting from amplification of an expanded set of 384 polymorphic *Pst* genes [36] (Additional file 1: Table S3). Each dataset was analysed using an automated MARPLE diagnostics pipeline [36] that included: (i) alignment to the *Pst* reference gene sets (isolate PST-104E [37]) with BWA version 0.7.5 [28] (ii) variant calling using SAMtools version 0.1.19 [38], (iii) generation of consensus gene sequences as described previously [31], (iv) sequence alignment, and (v) phylogenetic analysis using the GTRGAMMA model in RAxML version 8.2.12 [39]. Sequence alignments for each *Pst* sample were interrogated to determine the number of genes with at least 10% breadth of coverage, that were then classified as successfully amplified. Association between successful gene amplification and gene length were examined using custom Python and R scripts [32].

### *Pgt* marker gene selection

A total of 1,611 *Pgt* genes of 1–2 Kb in length and harbouring > 0.025 SNPs/base across the 86 *Pgt* isolates were selected from the *Pgt* reference genome for analysis (CRL 75-36-700-3 [27]) (Additional file 1: Table S4). Consensus gene sequences were generated for each of the 86 global *Pgt* isolates by independently incorporating sequence variation for each *Pgt* isolate into the 1,611 *Pgt* genes as described previously [31]. Genes were extracted from this 1,611 gene set, that fulfilled the threshold of 0.10 to 0.19 SNPs/bp at 0.005 increments across all 86 *Pgt* isolates. In addition, for each SNPs/bp threshold six subsets of genes were randomly generated using a selection probability that was length-dependent and determined using:

$$P\left( {chosen} \right) = 0.2 - \frac{{0.6 \times \:\left( {L - 1500} \right)}}{{1000}};{\rm{ }}L \ge 1kb$$

After estimating the selection probability per gene, six gene subsets were randomly chosen that each comprised of approximately 60% of the total gene number at each SNPs/bp threshold. The consensus gene sequences of the seven gene lists per SNPs/bp threshold were then used to generate phylogenetic trees or cladograms using an approximate maximum likelihood approach with FastTree version 2.1.9 [33]. To assess bipartitions of *Pgt* isolates at each SNPs/bp threshold the Robinson-Foulds metric was used [40]. This analysis enables an assessment of deviations in the distance between tree branches and was implemented using the TreeCMP software [41] with the RF distances halved [RF(0.5)] to determine changes in relation to the reference phylogeny.

### Primer design and pooling strategy

Primers were designed for amplification of the 392 initial *Pgt* marker genes using Primer3 software [42] returning 10,000 primer pairs between 15–30 base length, with an optimum annealing temperature of 60 ^o^C. Primer annealing sites were assessed within 500 bases of the 5’ and 3’ ends of the gene coding region, with primers iteratively tested for specificity starting with those closest to the coding sequence, ensuring amplification of the entire gene while minimising redundant bases from the 500 bp padding regions. Primer pairs were further assessed to prevent non-specific amplification in the plant host (IWGSC RefSeq v2.1 [43]) and for conservation across a genetically diverse array of *Pgt* isolates: CRL 75-36-700-3 [27], 99KS76A [44], 21 − 0, Ug99 [21] and UK-01 [23]. A custom Python script was used to identify and discard primer pairs with less than 3 mismatches with the plant host genome or more than 3 mismatches with any of the five *Pgt* reference genomes [32]. Following these criteria, a total of 276 primer pairs were successfully designed and allocated to four discrete primer pools using PrimerPooler v1.88 [45], which uses a ΔG cut-off of -7 kJ for prevention of dimer formation between primers in each pool.

### Annotation of the 276 *Pgt* marker genes

The selected 276 *Pgt* gene sequences were extracted from the *Pgt* reference gene set (CRL 75-36-700-3 [27]) and chromosome locations determined in the chromosome-level *Pgt* reference genome (isolate RKQCC [22]) through BLAST sequence similarity searches, with ≥ 95% match and > 1,000 bit-score [32]. Gene ontology (GO) term enrichment analysis was performed using GOATOOLS version 1.4.12 [46] with the GO-basic ontology, considering second-level terms and above, and a *p*-value cutoff for overrepresentation of < 0.05. Protein sequences of the 276 *Pgt* genes were assessed for secretion signals using SignalP3 and default settings [47]. Transmembrane domain containing proteins and proteins with mitochondrial signal peptides were removed using TMHMM and TargetP, respectively. The remaining 39 predicted secreted *Pgt* proteins were analysed using EffectorP 3.0 to identify potential cytoplasmic or apoplastic effectors [48].

### Analysis of *Pgt*-infected field samples collected in Kenya and Ethiopia

*Pgt*-infected wheat leaf samples were collected in Tipis (Central rift region), Timau (Mt. Kenya region), Mau Narok (Central rift region) and Kithithina (Mt. Kenya region) in Kenya, and Alkaso, Meti, Waro Kolobo, Buyo Kecheme and Ambo in Ethiopia, and stored in RNAlater^®^ to preserve nucleic acid integrity (Additional file 1: Table S5). Approximately 10–20 mg of leaf tissue was disrupted in 200 µL of lysis buffer [0.1 M Tris-HCl pH 7.5, 0.05 M ethylenediaminetetraacetic acid (EDTA) pH 8 and 1.25% sodium dodecyl sulphate (SDS)] using a micropestle. After settling at room temperature, 150 µL of supernatant was extracted, mixed with 360 µL of binding buffer PB (Qiagen, Manchester, UK) and DNA purified from solution using 30 µL of SeraSil-Mag™ 400 magnetic beads (Cytiva, Washington, USA). Following incubation for approximately 5 m on a magnetic rack, the clear supernatant was discarded and remaining pellet washed twice with 80% ethanol. DNA was recovered using 30 µL of elution buffer EB (Qiagen, Manchester, UK). The 276 polymorphic *Pgt* genes were amplified from each sample using multiplex PCR with the four primer pools and AmpliTaq Gold™ 360 Master Mix (Applied Biosystems, Massachusetts, USA). PCR conditions used were 95 ^o^C for 10 m, 40 cycles of 95 ^o^C for 15 s, 60 ^o^C for 30 s, and 72 ^o^C for 3 m, and a final extension at 72 ^o^C for 7 m. Resulting PCR products were purified using SeraSil-Mag™ 400 magnetic beads (Cytiva, Washington, USA) as above. PCR amplicons were prepared for nanopore sequencing on the MinION device using the library preparation kit SQK-RBK114.24 (Oxford Nanopore Technologies, Oxford, UK), following the manufacturer’s instructions. Samples were barcoded and sequenced on the MinION Mk1B using Flow Cells FLO-MIN114 R10.4.1 (Oxford Nanopore Technologies, Oxford, UK) until at least 250 million reads were generated per sample. Resulting datasets were basecalled using the MinKNOW software (Oxford Nanopore Technologies, Oxford, UK).

Bioinformatic analysis was implemented using a custom Snakemake [49] workflow [36]. This makes use of: (i) FastQC version 0.12.1 and Nanoq version 0.10 [50] for quality control and filtering of the nanopore reads, (ii) BWA version 0.7.18 [28] for sequence alignment, (iii) gffread version 0.12.7 [51] to identify gene locations in the reference genome annotations, (iv) SAMtools version 1.20 [28] for read-mapping statistics, indexing the reference genomes and generating pileup information, and (v) FastTree version 2.1.11 [33] for generation of phylogenetic trees. MultiQC version 1.23 [52] was incorporated into the Snakemake workflow to generate reports per sample that includes read quality and mapping statistics. All sequencing and bioinformatic analysis were performed locally on a 16’ PCSpecialist RECOIL^®^ laptop (16-Thread Intel i7-11800; NVIDIA GeForce RTX 3080 Max-Q; Wakefield, UK).

### Integration of fungicide target genes into the *Pgt* MARPLE diagnostics platform

Fungicide target genes (*Cyp51*, *SdhA*, *SdhB*, *SdhC* and *SdhD*) were extracted from the annotated *Pgt* reference genome (isolate Pgt21-0 [21]). To identify conserved regions suitable for primer design, BLAST sequence similarity searches were conducted to identify the genes in the four additional *Pgt* reference genomes: CRL 75-36-700-3 [27], 99KS76A [44], Ug99 [21], UK-01 [23]. Primers were designed using Primer3 software [42], with an optimum annealing temperature of 60 ^o^C. To generate DNA for initial testing of the PCR primers, *Pgt* infection assays were conducted. Three *Pgt* isolates (UK-01, UK-03 and UK-13) were used for inoculation of the hexaploid wheat (*Triticum aestivum* L.) varieties Siskin or Vuka, following methods described previously [53]. DNA was extracted from *Pgt*-infected leaf samples and each primer pair assessed using PCR conditions identical to those used to amplify the 276 polymorphic *Pgt* gene set. Subsequently, primers were incorporated into each of the four MARPLE gene pools and amplification of the five fungicide target genes assessed by PCR using DNA extracted from wheat varieties Siskin or Vuka infected with *Pgt* isolates UK-03 or UK-13.

Non-synonymous mutations reported to impact fungicide sensitivity in other fungal plant pathogens were identified through literature searches for *Zymoseptoria tritici*,* Blumeria graminis* f. sp. *tritici*,* Puccinia triticina*, *Pyrenophora teres*, and *Phakopsora pachyrhizi* (Additional file 1: Table S6) [54]. Amino acid sequence alignments were conducted with *Pgt* and the aforementioned pathogens for *Cyp51* and the three SDH complex subunits (*SdhB*, *SdhC* and *SdhD*) that tend to harbour mutations linked to fungicide resistance [10] using CLUSTAL W version 2.1 [55]. Custom Python scripts were developed to (i) highlight any analogous non-synonymous mutations in *Pgt Cyp51*, *SdhB*, *SdhC* and *SdhD* and (ii) search for any novel mutations across the five *Pgt* reference genomes (CRL 75-36-700-3 [27], 99KS76A [44], 21 − 0, Ug99 [21] and UK-01 [23]). The scripts were developed to also account for variation between haplotypes for each fungicide target gene within the *Pgt* reference isolate 21 − 0 (Additional file 1: Table S7). Python scripts were incorporated into the Snakemake workflow in the *Pgt* MARPLE diagnostics pipeline [36].

## Results

### Global *Pgt* isolates are highly diverse and group into 12 distinct genetic groups

**Fig. 1:**
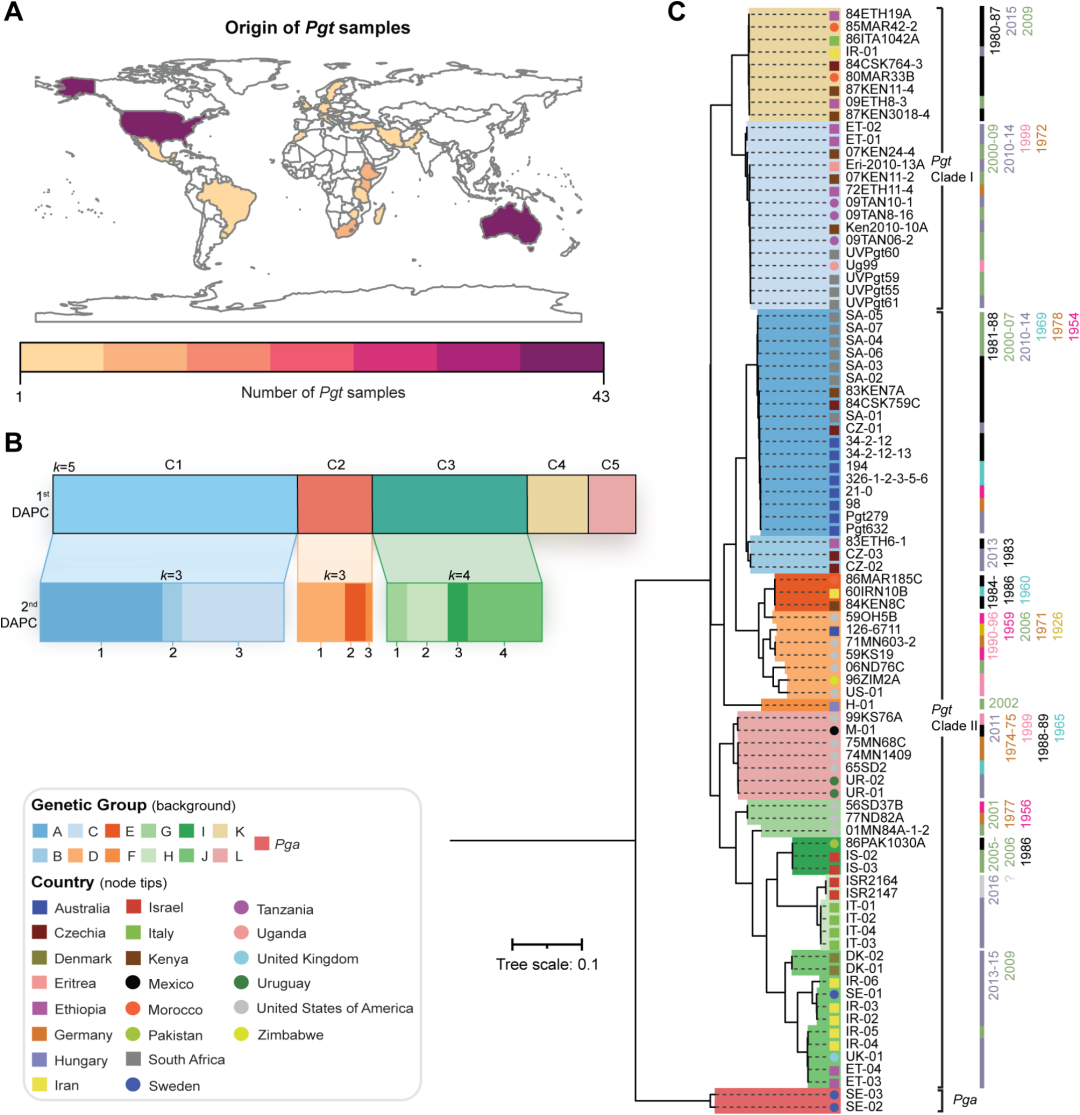
*Pgt* isolates collected across 25 countries are highly diverse, grouping into 12 distinct genetic groups. (**A**) Genomic and transcriptomic data were collated from 165 *Pgt* samples that were collected across 25 countries, with the largest number of *Pgt* samples originating from North America and Australia. (**B**) A subset of 86 global *Pgt* isolates underwent multivariate discriminant analysis of principal components (DAPC), identifying 12 distinct genetic groups (labelled A-L). For this analysis, 19,250 biallelic single nucleotide polymorphism (SNP) sites were utilised. Initial DAPC analysis based on assessment of the Bayesian Information Criterion (BIC) separated the *Pgt* isolates into five major genetic clusters (1st). However, the high diversity of the global *Pgt* population limited resolution within groups with high within-group variation. A second DAPC analysis was thus performed on population groups with > 10 *Pgt* isolates (2nd) to improve clustering resolution. (**C**) The global *Pgt* population analysed consisted of 12 genetically distinct groups. Phylogenetic relationships were inferred using an approximate maximum-likelihood model based on 86 genomic or transcriptomic *Pgt* datasets, along with two *Puccinia graminis* f. sp. *avenae* (*Pga*) genomic datasets used as an outgroup. Scale bar indicates the mean number of nucleotide substitutions per site

To assess the genetic variation across available *Pgt* genomic and transcriptomic datasets we gathered a collection of 165 global *Pgt* datasets for analysis (Fig. 1a). These included datasets derived from *Pgt* isolates collected across 25 countries from 1926 to 2016 [21–26] (Additional file 1: Table S1). Following quality filtering, each dataset was aligned to the *Pgt* reference genome (CRL 75-36-700-3 [27]) with an average of 81.4% (S.D. ±11.4%) of reads from genomic datasets and 70.2% (S.D. ±21.2%) reads from transcriptomic datasets aligned (Additional file 1: Table S2). To ensure each dataset originated from a single *Pgt* genotype we determined the distribution of read counts for biallelic single nucleotide polymorphisms (SNPs); as *Pgt* is a dikaryon it is expected that SNPs will have a single mode at 0.5 in heterokaryotic positions. This analysis highlighted 15 *Pgt* datasets with modes deviating from the normal distribution at 0.5 that were removed from subsequent analyses (Additional file 1: Table S1 and Additional file 2: Fig. S1). We also removed an additional set of 46 *Pgt* datasets that lacked coverage for an average of 13,909 genes (S.D. ±2,344 genes) out of a total of 16,309 predicted genes in the *Pgt* reference genome (CRL 75-36-700-3 [27]) (Additional file 3: Fig. S2).

To assess the genetic similarity between the remaining 86 *Pgt* isolates we conducted phylogenetic analysis and multivariate discriminant analysis of principal components (DAPC), with two *Puccinia graminis* f. sp. *avenae* (*Pga*) isolates included in the phylogeny as outliers (Fig. 1b-c; Additional file 1: Table S1). In the DAPC analysis, a range of *k*-values from 3 to 15 were analysed (Additional file 4: Fig S3a), with assessment of the Bayesian Information Criterion (BIC) indicating *k* = 5 as the optimum clustering solution, based on the lowest associated BIC value (Fig. 1b; Additional file 4: Fig. S3b). However, we previously noted that when analysing large, highly diverse rust pathogen populations using DAPC analysis, two iterations of DAPC were required; the first to resolve populations with high levels of genetic differentiation and the second to resolve lower levels of within-group variation present in the initial higher-level population clusters [19]. Following this strategy, we conducted a second round of DAPC analysis on three of the initial population clusters that had more than 10 *Pgt* individuals to further investigate any additional within-group genetic substructure. Assessment of the BIC values and cross-comparison with the phylogenetic analysis highlighted that the 3 initial population clusters could be further subdivided (Fig. 1b; Additional file 4: Fig. S3c); clusters 1 and 2 were subdivided into 3 further groups, and cluster 3 into 4 groups.

In the phylogeny, all *Pgt* isolates were separated between two major clades as reported previously [56], with further subdivisions coinciding with the 12 distinct global *Pgt* population clusters designated as genetic groups A – L (Fig. 1b-c). *Pgt* isolates also clustered largely based on year of emergence, with apparent examples of strain resurgence in modern times (Fig. 1c; Additional file 1: Table S1). For instance, genetic group K is mostly populated with *Pgt* isolates from 1980 to 1987, with two additional *Pgt* isolates collected in 2009 and 2015. We also noted that only two of the genetic groups (C and L) contained *Pgt* isolates collected in a single continent, with the largest group (C, 15 *Pgt* isolates) containing isolates collected across Africa in Ethiopia, Uganda, South Africa, Kenya, Eritrea, Tanzania and Ethiopia between 1972 and 2014 [21, 22]. This included *Pgt* isolates from the *Pgt* Ug99 race group [21]. The remaining 9 genetic groups contained *Pgt* isolates that were collected across continental boundaries, with the largest of these genetic groups (A, 18 *Pgt* isolates) including *Pgt* isolates collected over a 60-year period from 1954 to 2014 in Australia, Czechia, South Africa, Ethiopia and Kenya. The earliest *Pgt* isolates included in the analysis were contained in genetic group D, that contained *Pgt* isolates from Australia, USA and Zimbabwe, collected from 1926 (Australia *Pgt* isolate 126–6711) to 2006 (USA *Pgt* isolate 06ND76C). Overall, this analysis indicates that this collection of global *Pgt* isolates is highly diverse, with genetically related *Pgt* isolates also highly geographically dispersed.

### A subset of highly polymorphic genes can be used to define *Pgt* lineages

**Fig. 2:**
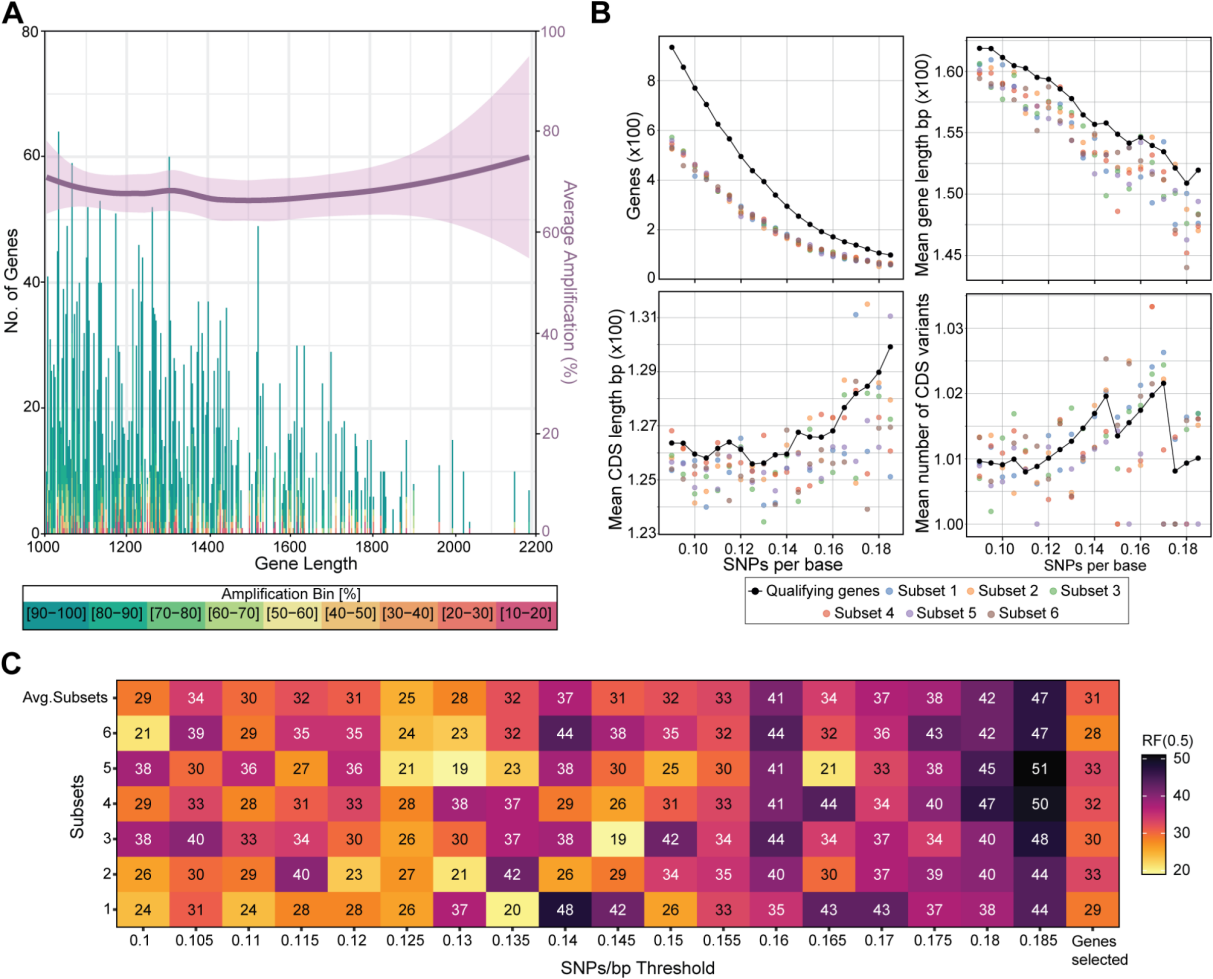
A subset of 276 *Pgt* genes, ranging from 1 to 2 Kb in length and exhibiting 0.125–0.130 SNPs/bp, can be used to accurately define *Pgt* lineages. **(A)** Data obtained from amplicon re-sequencing of *Puccinia striiformis* f. sp. *tritici* (*Pst*) genes revealed an average amplification success rate of over 60%, which decreased inversely with gene length. A total of 18 *Pst* amplicon datasets were evaluated from two available *Pst* marker gene sets: (i) 10 from the original set of 242 *Pst* marker genes [19], and (ii) 8 from an expanded and updated set of 384 *Pst* marker genes [36]. Purple line and shading indicates mean percentage and standard deviation of the gene amplified. **(B)** Analyses was conducted on the number of genes, length of genes and coding sequences (CDS) and number of CDS variants for *Pgt* genes at varying SNPs/bp thresholds. A total of 1,611 *Pgt* genes, ranging from 1 and 2 kb in length and exhibiting a minimum of 0.025 SNPs/bp variation between the 86 *Pgt* isolates, were selected for comparative analysis. Additionally, six subsets (Subsets 1–6) comprising of genes with a 60% mean probability of being included for each SNPs/bp threshold per *Pgt* isolate were randomly chosen and analysed to account for the potential 40% failure rate in amplification and/or sequencing observed in the *Pst* analysis. **(C)** Assessment of the Robinson-Foulds (RF) distance for comparing diversity between phylogenetic trees, indicated the lowest RF value ranged from 0.12 to 0.13 SNPs/bp. RF distance analysis was performed between the phylogenetic tree including 100% of the genes and the six trees with gene subsets at each SNP/bp threshold. The smallest difference was observed in the 0.125–0.130 SNPs/bp sets, which included 392 genes, of which 276 were successfully amplified (referred to as “genes selected”)

To reduce the amount of data generated per *Pgt* isolate, and thereby make it suited for amplicon-based sequencing on the MinION nanopore sequencing device, we set out to identify a subset of highly polymorphic *Pgt* genes that could be used to distinguish *Pgt* lineages in the phylogeny. A similar approach had been used previously to select a subset of polymorphic *Pst* genes suitable for amplicon-based nanopore sequencing [19] that were subsequently expanded [36]. Thus, we decided to conduct an evaluation on the established *Pst* polymorphic gene set to determine if any information could be gained to better guide *Pgt* gene selection. We examined 18 amplicon datasets generated from two available *Pst* marker gene sets: (i) 10 from the original 242 *Pst* marker genes [19], and (ii) 8 from an expanded and updated set of 384 *Pst* marker genes [36]. We found that on average 36% (S.D. ±12.0%) of *Pst* genes lacked coverage in the MinION datasets generated (Fig. 2a; Additional file 1: Table S3). In addition, we noted that *Pst* genes of > 2 kb in length were underrepresented in datasets following MinION sequencing (Fig. 2a). This indicates that a minimum of 40% redundancy and maximum gene length of 2 kb would be advantageous to consider when selecting genes for inclusion in the *Pgt* marker gene set.

To identify subsets of highly polymorphic *Pgt* genes that could be used to define *Pgt* isolates into the lineages in the phylogeny, we selected a total of 1,611 *Pgt* genes that were between 1 and 2 kb in length and had a minimum of 0.025 SNPs/bp variation between the 86 *Pgt* isolates, for comparative analysis (Additional file 1: Table S4). For each of the 86 global *Pgt* isolates we generated consensus gene sequences by independently incorporating sequence variation for each *Pgt* isolate into the 1,611 *Pgt* genes. We selected subsets of genes from the 1,611 genes with an average minimum of 0.10 to 0.19 SNPs/bp at 0.005 increments across all 86 *Pgt* isolates, which represented 767 to 90 polymorphic genes (Fig. 2b). We also randomly selected subsets of approximately 60% of genes at each SNPs/bp threshold per *Pgt* isolate to account for the potential for a 40% failure rate in amplification and/or sequencing as observed with *Pst* (Fig. 2b). Phylogenetic trees were generated using these gene subsets and compared to phylogenies generated using the full gene set at each SNPs/bp threshold (Additional file 5: Fig. S4). To assess deviation between phylogenies generated using the gene subsets and full gene sets we analysed the bipartitions of the branches separating the *Pgt* isolates using the Robinson-Foulds (RF) distance [40]. This analysis indicated that the lowest RF value was between 0.12 and 0.13 SNPs/bp, resulting in selection of 392 highly polymorphic *Pgt* genes (Fig. 2c).

### Sequences of 276 highly polymorphic *Pgt* genes are sufficient for distinguishing *Pgt* lineages

**Fig. 3:**
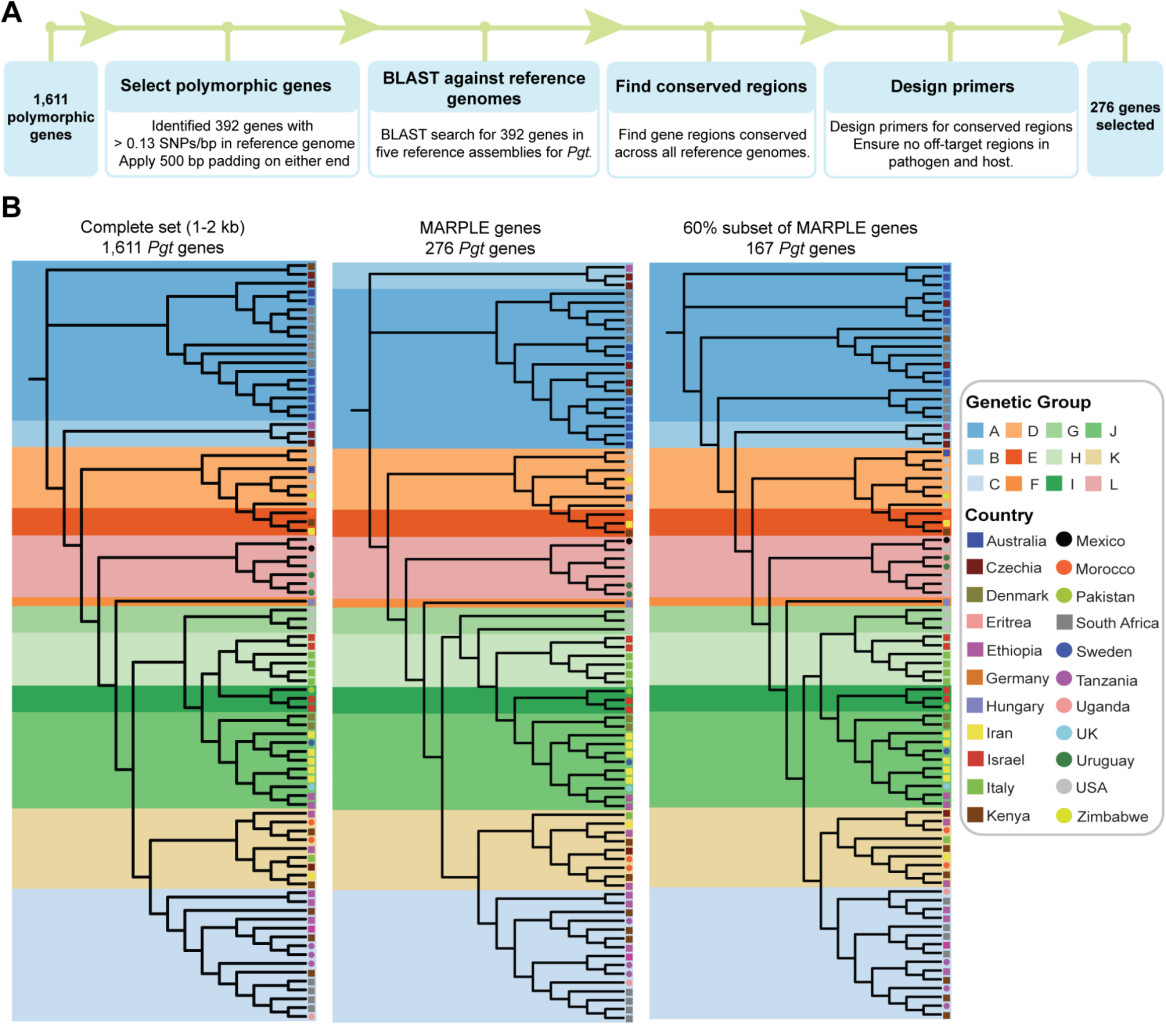
A collection of 276 highly polymorphic *Pgt* genes is sufficient to accurately reconstruct the phylogeny generated using the complete set of 1,611 polymorphic *Pgt* genes. (**A**) Primers were successfully designed for 276 highly polymorphic *Pgt* genes for use in the *Pgt* MARPLE diagnostics platform. Starting with an initial pool of 1,611 *Pgt* genes (ranging from 1 to 2 kb in length, with a minimum of 0.025 SNPs/bp), a set of 392 highly polymorphic *Pgt* genes was selected and assessed for conservation across *Pgt* isolates and specificity, resulting in successfully designed primers for 276 of these *Pgt* genes. (**B**) The topology of the *Pgt* phylogeny was consistent whether constructed using the initial 1,611 *Pgt* genes, the 276 highly polymorphic *Pgt* genes, or a subset that accounted for a potential 40% failure rate in amplification and/or sequencing. Cladograms were generated using 86 *Pgt* isolates and an approximate maximum-likelihood model

To generate amplicons for re-sequencing on the MinION platform, we set out to design primers for amplification of the 392 highly polymorphic *Pgt* genes (Fig. 3a). Primer sequences were assessed in silico for sequence conservation across a series of genetically diverse *Pgt* isolates where genome assemblies are publicly available. This included: (i) *Pgt* isolate CRL-75-36-700-3 that was used as the reference genome and was collected in Pennsylvania (USA) in 1975 (pathotype SCCL [27]), (ii) *Pgt* isolate 21–0 that originated from Australia [21]), (iii) *Pgt* isolate UK-01 that was collected in the UK in 2013 (pathotype TKTTF [23]), (iii) *Pgt* isolate 99KS76A-1 collected in Kansas (USA) in 1999 (pathotype RKQQC [44]), and (v) a *Pgt* isolate belonging to the Ug99 race group collected in Uganda in 1999 (pathotype TTKSK [21]). To also evaluate the potential for non-specific amplification of host plant DNA, primer specificity was checked against the Chinese Spring wheat genome (IWGSC RefSeq v2.1 [43]). This led to robust primers being successfully designed for a total of 276 highly polymorphic *Pgt* genes (Additional file 1: Table S8). The resulting 276 *Pgt* gene set was then used to reconstruct the *Pgt* phylogeny, including RF analysis at a 40% potential failure rate (Figs. 2c and 3b). These results confirmed that 276 highly polymorphic *Pgt* genes were sufficient to reconstruct the topology of the full *Pgt* phylogeny, even when only approximately 60% of gene sequences are included.

### The selected 276 *Pgt* genes are evenly distributed in the *Pgt* genome and largely encode proteins with unknown function

**Fig. 4:**
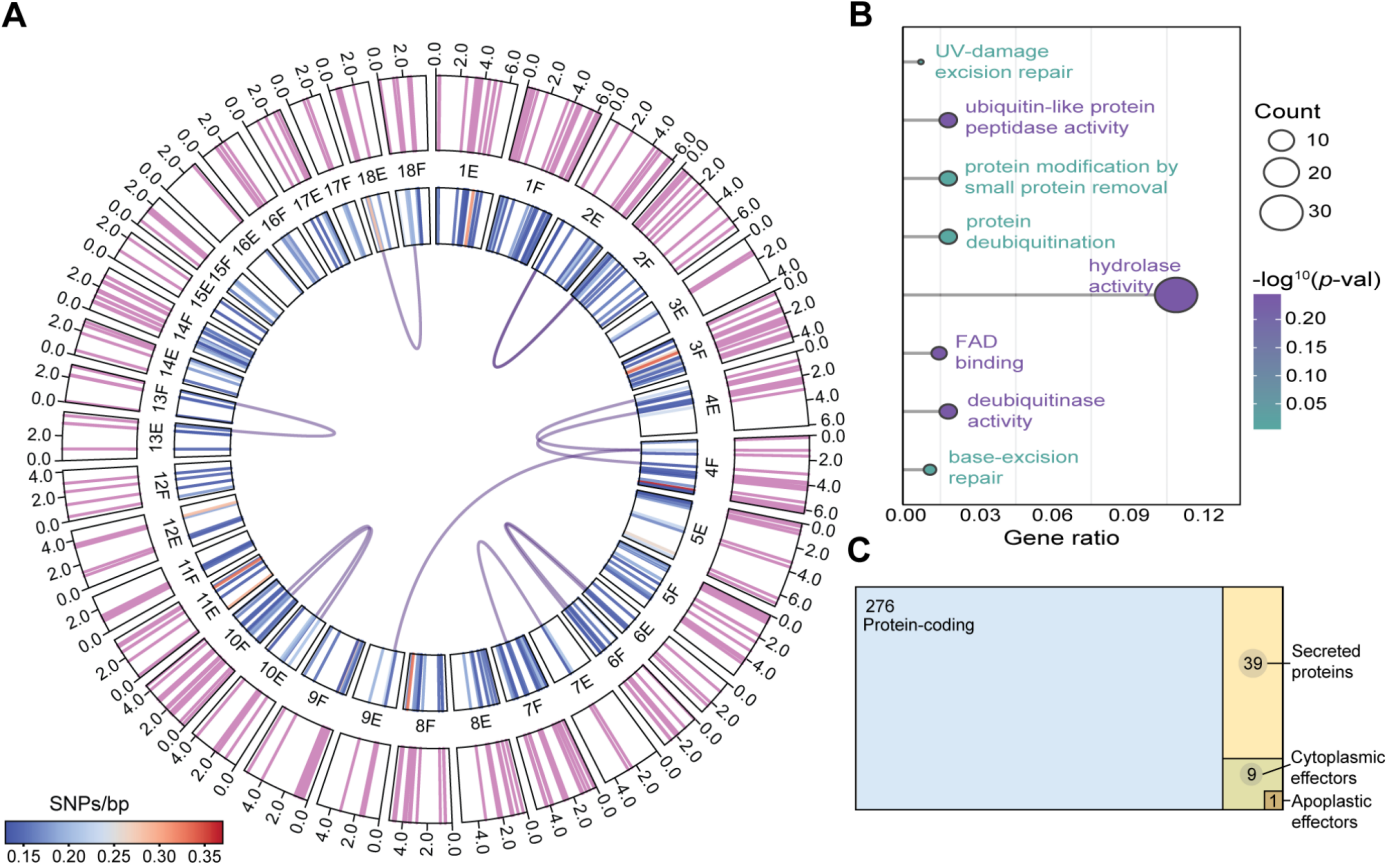
Annotation of 276 highly polymorphic *Pgt* genes showed uniform distribution across the *Pgt* genome, with proteins primarily enriched for hydrolase activity and few predicted effector proteins. (**A**) The selected 276 *Pgt* genes were distributed across all chromosomes (indicated by pink marks in the outer panel), showing no clustering based on average SNP/bp content for each gene (as represented in the inner colour-scaled panel). The physical location of the 276 highly polymorphic genes was examined across the ~ 180 Mb chromosome-level haplotype resolved *Pgt* reference genome (isolate RKQCC [22]). Lines connecting the chromosomes indicate locations of gene variants; E and F, haplotype chromosomes. (**B**) Gene Ontology (GO) enrichment analysis of the corresponding 276 predicted proteins indicated significant enrichment for hydrolase activity (GO:0016787) among the 89 annotated proteins. (**C**) Among the 276 *Pgt* genes, 39 encoded predicted secreted proteins, including one predicted to act as an apoplastic effector protein and nine as cytoplasmic effector proteins, with one of the putative cytoplasmic effectors predicted as dual localised. Protein sequences of the 276 *Pgt* genes were assessed for secretion signals using SignalP3 and default settings [47], with transmembrane domain containing proteins and proteins with mitochondrial signal peptides removed using TMHMM and TargetP, respectively. The 39 putative secreted *Pgt* proteins were analysed for predicted localisation using EffectorP 3.0 [48]

To assess the distribution of the 276 highly polymorphic genes across the *Pgt* genome, we examined their physical location in the available ~ 180 Mb chromosome-level haplotype resolved *Pgt* reference genome (isolate RKQCC [22]). This analysis indicated that the 276 *Pgt* genes were distributed across all 36 chromosomes, with an average of 7 genes per chromosome (Fig. 4a). We identified 9 genes that had > 99% identity and a bit-score > 1,000, in their matching haplotype chromosomes (E and F) and one gene that appeared in two unrelated haplotype chromosomes (chromosomes 9E and 4 F), potentially indicating a recent gene duplication (Fig. 4a). The largest number of genes (20 genes) were found on the largest 6.64 Mb chromosome (number 1). Chromosome 11 harboured genes with the highest level of overall diversity between *Pgt* isolates, exhibiting an average of 0.21 SNPs/bp (S.D. ± 0.075 SNPs/bp). Overall, this analysis indicates that the selected 276 polymorphic *Pgt* marker genes are well distributed across the *Pgt* genome.

To assess the biological processes associated with the selected 276 *Pgt* genes, we assigned gene ontology (GO) terms to each corresponding protein. A total of 89 of the corresponding proteins could be annotated with second-level GO terms, that included nine molecular functions (GO:0003824, GO:0140657, GO:0005488, GO:0003774, GO:0060090, GO:0098772, GO:0005198, GO:0140110 and GO:0005215), two cellular functions (GO:0110165 and GO:0032991), and six biological processes (GO:0065007, GO:0009987, GO:0098754, GO:0051179, GO0008152 and GO:0050896) (Additional file 6: Fig. S5 and Additional file 1: Table S9). We also compared GO terms assigned to the 276 *Pgt* proteins and the remaining *Pgt* proteome, which highlighted a high level of enrichment in GO:0016787 (hydrolase activity) (*p*-value > 0.05) (Fig. 4b) [46]. We noted that 68% of the 276 *Pgt* genes encoded proteins with unknown function, which is a common feature of pathogen effector proteins that typically lack sequence similarity to known proteins [57]. To explore if any of the 276 *Pgt* marker genes could encode putative effector proteins, we examined each protein for a predicted secretion signal (Additional file 1: Table S10). This analysis identified 39 predicted secreted proteins, with 28% (11 out of 39) of these proteins predicted to harbour hydrolase activity. We then used machine learning to determine if any of these 39 secreted proteins were predicted as putative cytoplasmic or apoplastic effectors [48], finding one protein predicted to function as an apoplastic effector protein and nine as cytoplasmic effector proteins, with one of the putative cytoplasmic effectors predicted as dual localised (Fig. 4c). Overall, this indicates that very few of the 276 highly polymorphic genes selected as markers encode proteins with a currently identifiable predicted function.

### MARPLE diagnostics can be used to rapidly assign *Pgt*-infected samples to known *Pgt* race groups in Kenya and Ethiopia

**Fig. 5:**
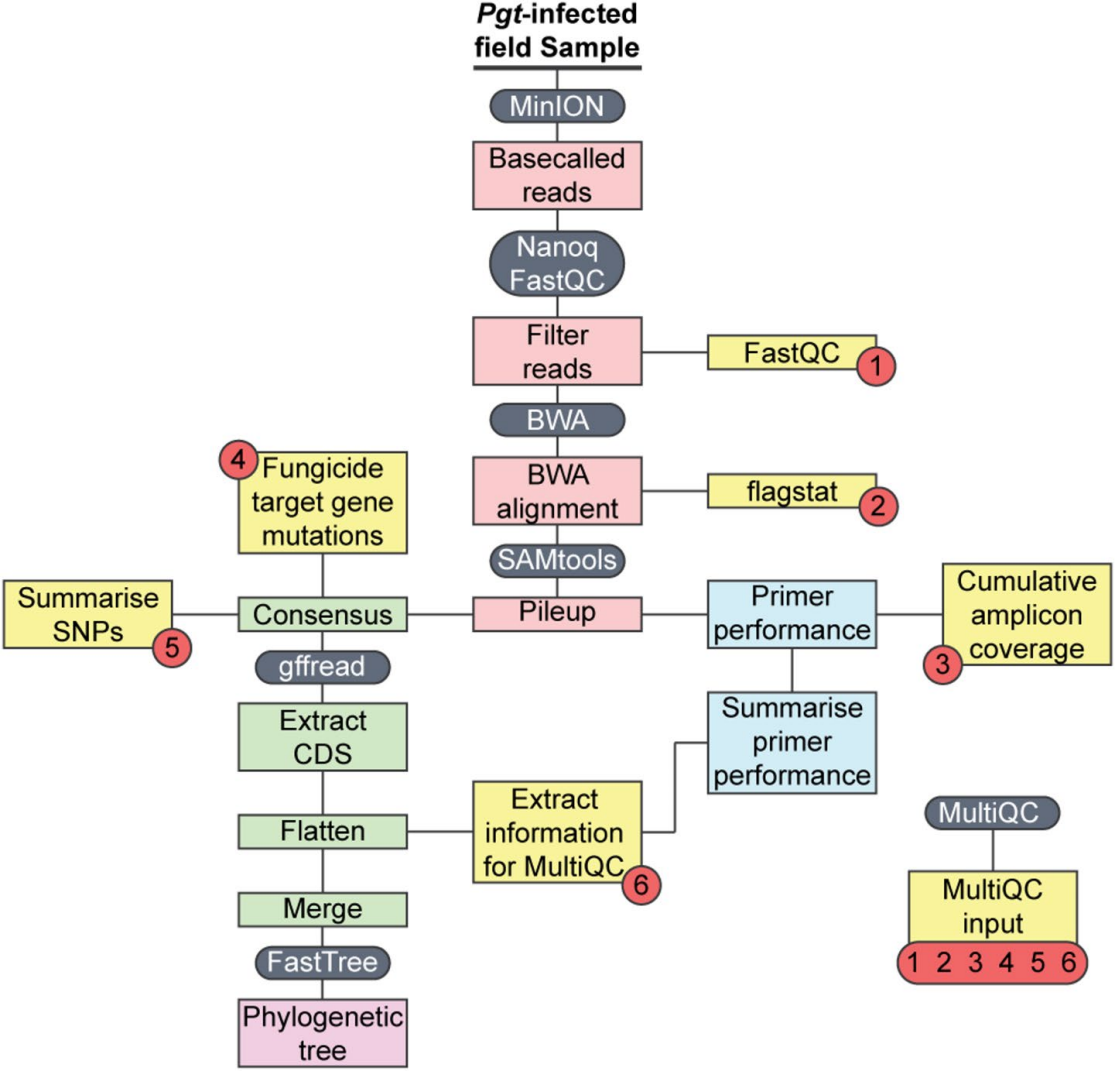
Overview of the *Pgt* MARPLE diagnostics bioinformatics Snakemake workflow. A schematic of the Snakemake workflow developed for the *Pgt* MARPLE diagnostics platform, which produces two primary outputs: (i) a phylogenetic tree and (ii) a MultiQC report. Red circles, numbered sources indicating data incorporated into the MultiQC report

To determine whether the 276 highly polymorphic *Pgt* marker genes are sufficient for genotyping unknown *Pgt* field samples in resource-limited locations we conducted a pilot study to assess the markers on *Pgt*-infected wheat samples collected in Kenya (5 samples) and Ethiopia (7 samples) (Additional file 1: Table S5). First, to generate an appropriate set of reference *Pgt* isolates for typing unknown *Pgt* isolates, we expanded our initial collection of 86 *Pgt* isolates to include publicly available datasets from an additional 16 *Pgt* isolates that had previously been typed to 9 major *Pgt* race groups, termed Clades I, II, III, IV, VI-A, VI-B, VI-C, VII, IX (Additional file 1: Table S11) [22]. *Pgt*-infected wheat samples were collected from fields in Kenya and Ethiopia in the 2022-23 wheat season and DNA extracted using a simple magnetic-bead-based extraction and purification method suited for use in resource-limited regions. Primers to amplify the 276 *Pgt* marker genes were then assembled into 4 pools to considerably reduce the number of PCR reactions required (Additional file 1: Table S8). Following PCR on a simple miniPCR machine, the resulting amplicons were purified and prepared for nanopore sequencing. Following basecalling, resulting datasets were analysed locally using a custom Snakemake data analysis workflow that includes extensive parallelisation [36] (Fig. 5). Implementation of this automated workflow resulted in MultiQC quality reports and phylogenetic trees being generated in less than 30 min in Kenya and Ethiopia, compared to approximately 20 h for the previous *Pst* MARPLE workflow [19].

**Fig. 6:**
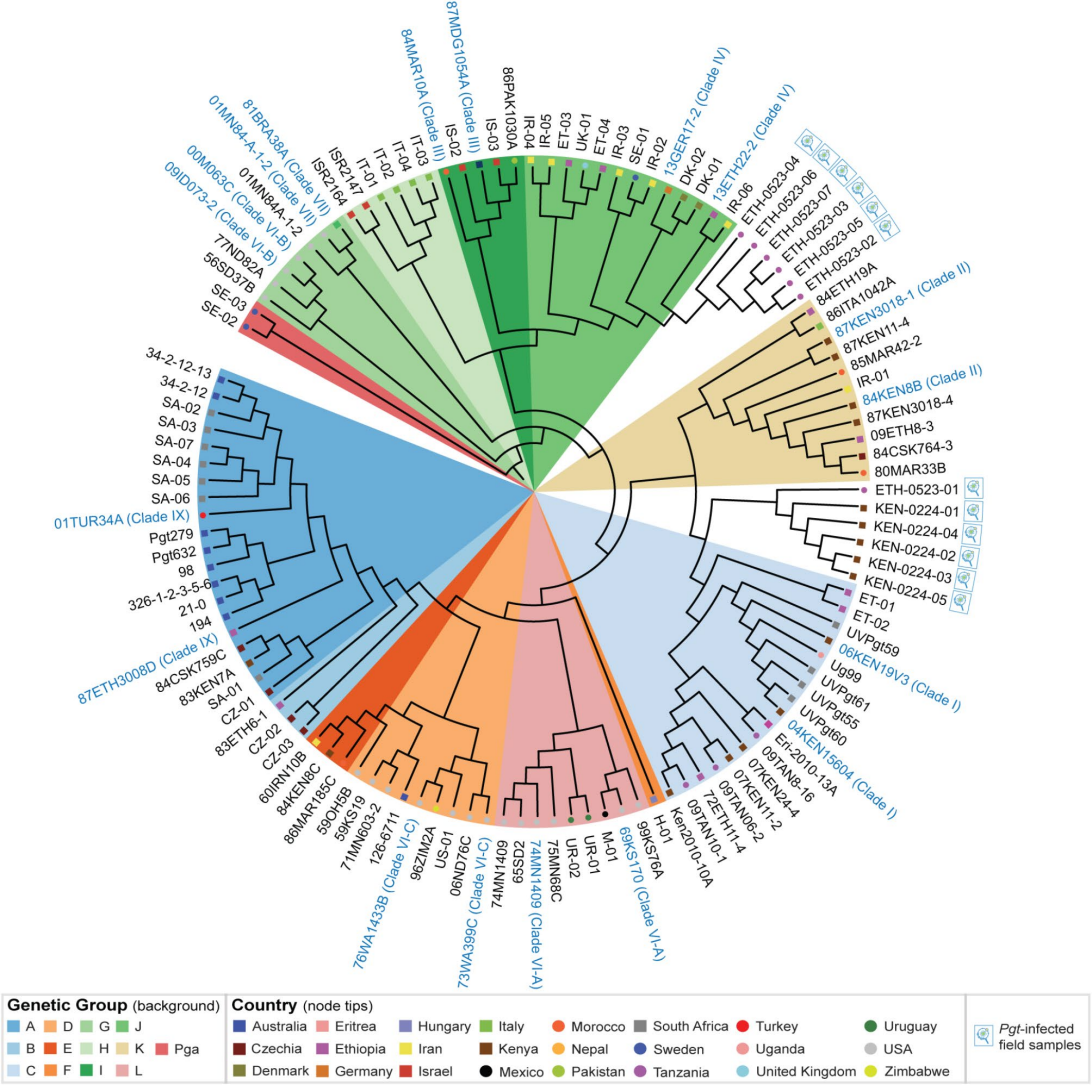
Genetic analysis of *Pgt*-infected field samples from Kenya and Ethiopia demonstrated close genetic relationships with *Pgt* representative isolates previously identified in East Africa. The *Pgt* MARPLE diagnostics platform was applied to *Pgt*-infected field samples in Kenya (5 samples) and Ethiopia (7 samples) to generate sequence data for the selected 276 highly polymorphic *Pgt* genes. The cladogram depicts evolutionary relationships inferred using an approximate maximum-likelihood model based on 102 genomic or transcriptomic *Pgt* datasets, which included 16 previously typed *Pgt* representative isolates classified into 9 major *Pgt* race groups, along with the 12 *Pgt* samples from Kenya and Ethiopia

To determine the efficiency of gene amplification for each *Pgt* field sample, we investigated the results within the inbuilt MultiQC report. This analysis indicated that all *Pgt*-infected samples surpassed the minimum 60% threshold of genes amplified and demonstrated a well-distributed amplification of genes across each primer pool: average amplification of genes in pool 1 80% (S.D. 8%), pool 2 81% (S.D. 6%), pool 3 76% (S.D. 8%) and pool 4 74% (S.D. 8%) (Additional file 7: Fig. S6). Phylogenetic analysis to compare the unknown *Pgt*-infected field samples to known *Pgt* representative isolates, confirmed all unknown *Pgt* samples belonged to race groups previously recognised as present East Africa (Fig. 6). For instance, *Pgt* samples collected in Kenya grouped in the phylogeny with *Pgt* isolates previously assigned to “Clade I”, which contains the Ug99 race group that is known to be the dominant race group in the region [21]. Overall, this analysis confirmed that the 276 highly polymorphic *Pgt* marker genes were sufficient for accurately genotyping unknown *Pgt* samples and that the MARPLE diagnostics pipeline could be successfully executed in resource-limited regions.

### Incorporating fungicide target genes into the *Pgt* MARPLE diagnostics platform

**Fig. 7:**
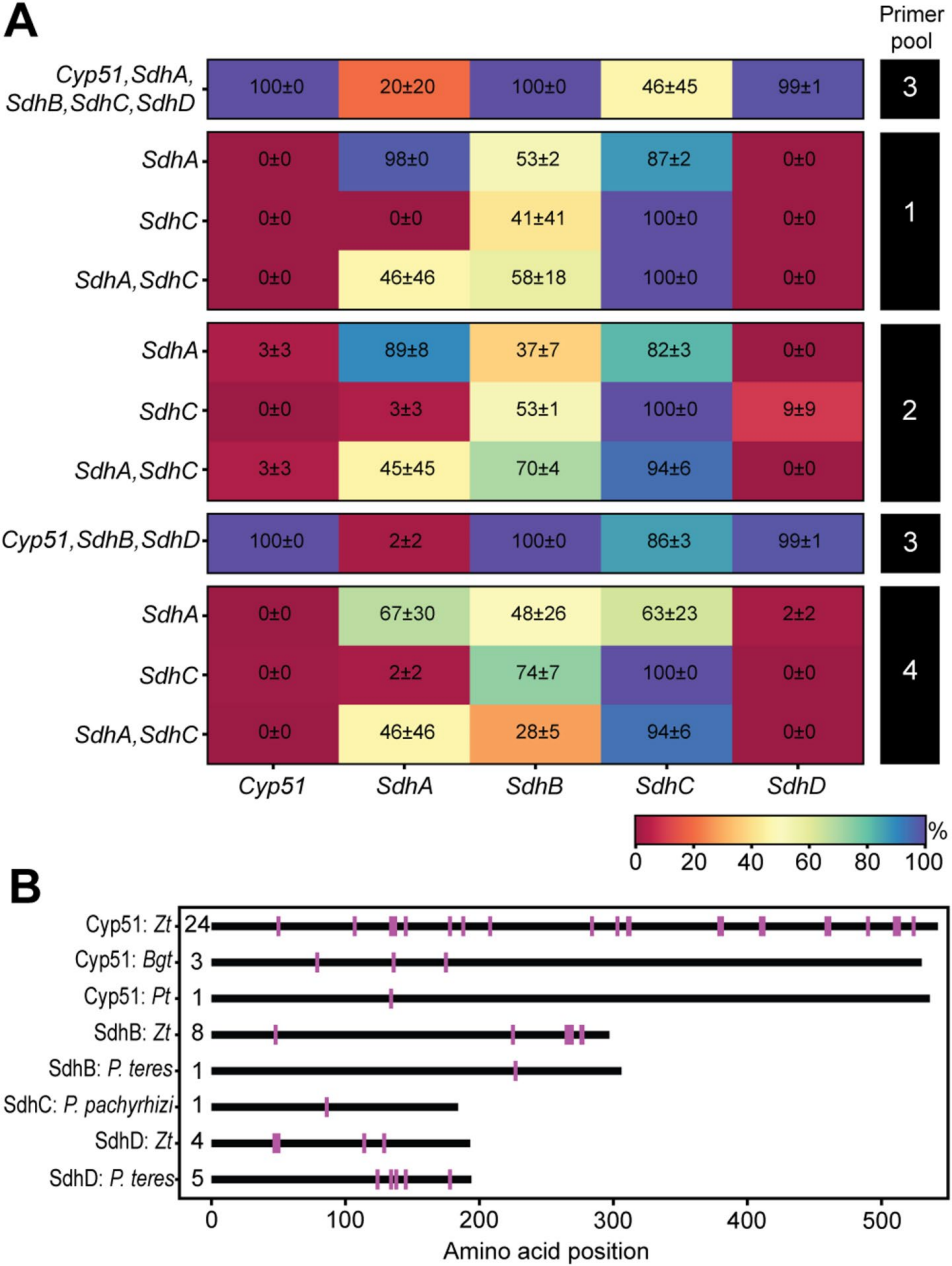
Optimising the pooling strategy for amplification of fungicide target genes in *Pgt* and highlighting resistance-associated mutations from other pathosystems for assessment in the MARPLE diagnostics platform. (**A**) To optimise amplification of the *Pgt* fungicide target genes (*Cyp51*, *SdhA*, *SdhB*, *SdhC* and *SdhD*), primer pairs were distributed across the four MARPLE diagnostics primer pools accordingly. Trials were conducted using DNA extracted from wheat material infected with *Pgt* isolates UK-03 and UK-13. Following gene sequencing, the average breadth of coverage was determined for each of the five genes, with the optimum breadth of coverage achieved when primers to amplify *SdhA* were added singly to pool 1, *Cyp51*, *SdhB* and *SdhD* to pool 3 and *SdhC* to pools 1, 2 or 4. (**B**) Non-synonymous mutations in *Cyp51*, *SdhB*, *SdhC* and *SdhD* that have been reported to influence fungicide sensitivity were collated for *Zymoseptoria tritici* (*Zt*), *Blumeria graminis* f. sp. *tritici* (*Bgt*), *Puccinia triticina* (*Pt*), *Pyrenophora teres* (*P. teres*), and *Phakopsora pachyrhizi* (*P. pachyrhizi*). This information was then incorporated into the MARPLE diagnostics Snakemake workflow to enable any analogous known mutations to be highlighted when processing *Pgt*-infected samples

To expand the utility of the *Pgt* MARPLE diagnostics platform during disease emergencies we decided to include the ability to rapidly identify mutations in known fungicide target genes that could be indicative of decreases in fungicide sensitivity. Orthologous sequences for *Cyp51*, *SdhA*, *SdhB*, *SdhC* and *SdhD* were extracted from the *Pgt* reference genome (isolate 21 − 0 [21]; Additional file 1: Table S7). As above, in silico analysis was conducted to design primers in regions conserved across the five *Pgt* reference genomes (CRL 75-36-700-3 [27], 99KS76A [44], 21 − 0, Ug99 [21], and UK-01 [23]), which were also not predicted to amplify in the wheat reference genome (IWGSC RefSeq v2.1 [43]). PCR reactions were conducted on DNA extracted from wheat infected with three *Pgt* isolates (UK-01, UK-03 and UK-13), confirming that the five primer pairs could successfully amplify all five target genes (Additional file 8: Fig. S7). To incorporate the primers into the *Pgt* MARPLE diagnostics system, all primer pairs were initially integrated into primer pool 3 for analysis. The efficiency of gene amplification was assessed using DNA extracted from wheat material infected with *Pgt* isolates UK-03 and UK-13. Following gene sequencing, the average breadth of coverage was determined for each of the five genes with *Cyp51* (100%, S.D. 0%), *SdhB* (100%, S.D. 0%) and *SdhD* (99%, S.D. 1%) achieving near-complete coverage (Fig. 7a). However, *SdhA* and *SdhC* had much lower breadths of coverage, 20% (S.D. 20%) and 46% (S.D. 45%), respectively. Therefore, we decided to assess the efficiency of the *SdhA* and *SdhC* primer pairs in each of the remaining 3 primer pools, independently and in combination. This analysis indicated that near-complete breadth of coverage could be achieved when the *SdhA* primer pair was incorporated singly into pool 1 (98%, S.D. 0%) and primers for *SdhC* in pools 1, 2 or 4 (singly 100%, S.D. 0% for each pool) (Fig. 7a). Thus, primers for amplification of *SdhA* were incorporated into primer pool 1, *Cyp51*, *SdhB* and *SdhD* into pool 3 and *SdhC* into pool 4 within the *Pgt* MARPLE diagnostics system (Additional file 1: Table S8).

As multiple fungal species also frequently gain the same mutation in response to the same chemical treatment [2], we collated information on known mutations in *Cyp51*, *SdhB*, *SdhC* and *SdhD* from *Zymoseptoria tritici*,* Blumeria graminis* f. sp. *tritici*,* Puccinia triticina*,* Pst*,* Pyrenophora teres*, and *Phakopsora pachyrhizi* (Additional file 1: Table S6) (Fig. 7b). For the wheat rusts, this includes the only currently reported non-synonymous mutation in *Cyp51* (Y134F) identified in *P. triticina* and *Pst* and the SdhC I85V mutation identified in *Pst* [54, 58]. This information was then incorporated into the MARPLE diagnostics Snakemake workflow to enable any analogous known mutations to be highlighted when processing *Pgt*-infected samples, alongside novel non-synonymous mutations that may be identified from alignment to the *Pgt* reference genes from *Pgt* isolate 21 − 0 [21] (Fig. 5; Additional file 9: Fig. S8).

## Discussion

Fungal plant disease outbreaks are increasing in scale and frequency, with recurrent *Pgt* epidemics capable of reducing wheat harvests by 10–70% annually across the globe [59]. Like other rust fungi, new *Pgt* strains are constantly emerging, and can rapidly disperse over vast distances via airborne dispersal of asexual urediniospores. This facilitates transmission of *Pgt* strains between regions, which can lead to widespread disease outbreaks as exotic *Pgt* strains often overcome resistance in dominant crop varieties in the new region [20]. For example, Ethiopia is the largest wheat producing country in Sub-Saharan Africa, and in the 2013–2014 wheat season the country suffered a devastating *Pgt* outbreak caused by influx of a ‘new’ *Pgt* race. The epidemic developed rapidly, resulting in yield losses of nearly 100% on the most widely grown wheat cultivar (Digalu), with national wheat production losses of at least 15% [60]. In other global regions, changes in agronomic practices and climate change are also impacting *Pgt* geographic distribution [53]. For instance, *Pgt* began to re-emerge in western Europe in 2013 and is now considered a burgeoning risk to wheat production in the region due to the high level of wheat vulnerability [61]; over 96% of UK wheat varieties were recently shown to be susceptible to *Pgt* infection [53]. Furthermore, the geographic distribution of the notorious *Pgt* race termed ‘Ug99’ also recently shifted, being reported in Nepal in 2023 [62]. Thus, there is an urgent need to develop a PoC, real-time *Pgt* genotyping platform that would enable early detection of newly emerging *Pgt* isolates.

To address this need, we developed a simplistic amplicon-based genotyping strategy to rapidly type individual *Pgt* strains using the portable MinION nanopore sequencing device. Following comparative genomic analysis of 86 global *Pgt* isolates, we identified a core set of 276 highly variable genes that we showed are informative for distinguishing individual *Pgt* lineages, even at a 40% failure rate in amplification. Annotation of these 276 genes indicated that 32% encode proteins with a known function, and revealed significant enrichment for proteins with hydrolase activity, when compared to the remaining *Pgt* proteome. Hydrolase activity was also common (28%) among proteins within this set that are predicted to encode secreted proteins. Plant pathogens secrete an array of hydrolytic enzymes during infection, that can act as virulence factors. For instance, serving a critical role in breaching the host cell wall, which acts as the main physical barrier against microbial attack [63]. Fungal peptidases also exhibit hydrolytic activity and can alter or deactivate protein components of the host defense machinery, ultimately suppressing defense responses [64]. This can result in significant nucleotide variability, as observed among genes encoding secreted peptidases in the fungal wheat pathogen *Zymoseptoria tritici* [64]. We also noted a higher proportion of genes predicted to encode secreted proteins among the selected 276 *Pgt* gene set (14%), than represented in the full *Pgt* proteome (6% [27]). This likely reflects the greater sequence variation found among *Pgt* genes that encode proteins with a virulence function.

In addition to increased virulence, sequence diversification can also play a significant role in the decline of fungicide sensitivity reported in many pathogenic fungi. Over time, environmental exposure to fungicides can result in resistance developing through mutations in genes encoding fungicide targets, overexpression or duplication of these target genes, overexpression of linked transporters encoding efflux pumps and/or epigenetic changes altering gene expression, among others [65]. Traditionally, identifying resistant individuals relied solely on lengthy bioassays that provided detailed analysis of resistance levels. However, rapid molecular detection methods are now increasingly being used to identify alleles of target genes associated with resistance, enabling quicker monitoring of breakdowns in fungicide sensitivity. Thus, within the *Pgt* MARPLE diagnostics platform we also incorporated the ability to detect mutations in genes encoding the targets of two major fungicide classes used to tackle wheat rust outbreaks: azoles and SDHIs [66]. Within this system we also integrated the ability to highlight any analogous mutations reported in *Z. tritici*,* B. graminis* f. sp. *tritici*,* P. triticina*,* Pst*,* P. teres*, and *P. pachyrhizi.* Given the extensive parallel evolution of similar point mutations across various fungal species [2], monitoring for these characterised resistance alleles from other pathogens within *Pgt* may offer an early warning of shifts in fungicide sensitivity. This would expedite follow-up detailed bioassays and adjustments in fungicide application regimes to help protect crop yields.

While molecular diagnostic techniques are highly effective, their application is often limited to well-equipped laboratories with abundant resources. In contrast, the *Pgt* MARPLE diagnostics platform was developed to be specifically suited for use in resource-limited locations that are often the hardest hit during disease epidemics. To achieve this, we further optimised the existing simplistic system we established previously for processing *Pst*-infected wheat samples [19] and automated the bioinformatics analysis by introducing the Snakemake workflow engine [67]. This improvement in the bioinformatic analysis, reduced the time to generate the phylogenetic trees from approximately 20 h in the original *Pst* MARPLE workflow [19] to under 30 min. The Snakemake workflow also now produces automated MultiQC quality reports, allowing users to rapidly access the quality of the data generated. By comparing unknown *Pgt* isolates within the MARPLE diagnostics system to data from 9 major *Pgt* race groups [22], samples can be promptly assigned to known *Pgt* lineages within less than 2 days of collecting samples. Additionally, any *Pgt* isolates found to be dissimilar to *Pgt* representatives from known race groups, that could represent new incursions, can be quickly identified and highlighted for further investigation. Implementation of the *Pgt* MARPLE diagnostics platform in Kenya and Ethiopia demonstrated its effectiveness in accurately defining *Pgt*-infected wheat samples, assigning newly tested samples to lineages previously reported in East Africa. As additional data for more *Pgt* race groups becomes available, the *Pgt* MARPLE diagnostics system will become even more robust in accurately assigning *Pgt*-infected samples.

## Conclusion

Effective management of fungal disease outbreaks hinges on prompt and accurate diagnostics to guide suitable treatment interventions. Over the past two decades, significant advances in genomic-based molecular diagnostic tools have created abundant new opportunities to enhance the speed and precision of disease diagnostic strategies. Despite these advances, implementing genomic-based diagnostics in resource-limited regions – often hardest hit by disease epidemics – remains challenging. However, the advent of the portable MinION sequencer has unlocked new possibilities for PoC disease diagnostics. By combining the portability of the MinION sequencer with amplicon-based genotyping to navigate the complexities of obligate fungal pathogens, we developed the near real-time *Pgt* MARPLE diagnostics platform. This platform offers the first portable system for strain-level diagnostics of *Pgt* directly from field-collected, *Pgt*-infected wheat samples, delivering results within 2 days of sample collection. Additionally, the platform’s capability to monitor mutations in fungicide target genes provides an early warning system for potential indicators of declines in fungicide sensitivity, allowing timely intervention. This study also provides a robust framework that can be adapted and applied to develop similar diagnostics and surveillance platforms for other complex fungal threats to plant health. As advanced genomic-based diagnostic tools are increasingly integrated into national surveillance programmes, our ability to manage disease outbreaks will improve, helping reduce the devastating losses currently caused by these ‘cereal killers’.

## Electronic supplementary material

Below is the link to the electronic supplementary material.

**Additional file 1**: **Tables S1-S10**. Microsoft Excel Workbook containing 10 worksheets. Table S1: Description of *Pgt* isolates analysed in this study. Table S2: Percentage of reads aligned to the *Pgt* reference genome assembly CRL 75-36-700-3. Table S3: Amplification of polymorphic genes in *Puccinia striiformis *f. sp.* tritici* (*Pst*) from *Pst*-infected wheat field samples. Table S4: The 1,611 *Pgt* genes selected between 1 and 2 kb in length that had a minimum of 0.025 SNPs/bp variation between the 86 *Pgt* isolates analysed. Table S5: *Pgt*-infected wheat field samples collected in Kenya and Ethiopia and analysed using the *Pgt* MARPLE diagnostics platform. Table S6: Non-synonymous mutations previously reported in the fungicide target genes (*Cyp51*, *SdhA*, *SdhB*, *SdhC* and *SdhD*) from various fungal plant pathogens. Table S7: Location of fungicide target genes in the Pgt21-0 reference assembly. Table S8: Primer pairs and pooling strategy for the 276 *Pgt* genes included in the MARPLE diagnostics platform. Table S9: Annotation of the 276 polymorphic *Pgt* genes included in the MARPLE diagnostics platform. Table S10: A total of 39 of the 276 polymorphic *Pgt* genes encoded predicted secreted proteins. Table S11: Details of the known pathotypes of 16 additional *Pgt* isolates that had previously been typed to 9 major *Pgt* race groups, termed Clades I, II, III, IV, VI-A, VI-B, VI-C, VII, IX.

**Additional file 2**: **Fig. S1**. Distribution of biallelic read counts for 165 *Pgt* isolates. Read frequency at single nucleotide polymorphisms (SNPs) for *Pgt* isolates that showed a normal distribution at 0.5 (A) and *Pgt* isolates with modes deviating from the normal distribution (B). A total of 165 *Pgt* transcriptomics and genomic datasets were independently aligned to the *Pgt* reference genome (CRL 75-36-700-3 [27]) and the distribution of read counts for biallelic SNPs assessed to ensure each dataset originated from a single *Pgt* genotype; as *Pgt* is a dikaryon it is expected that SNPs will have a single mode at 0.5 in heterokaryotic positions.

**Additional file 3**: **Fig. S2**. Assessment of read depth coverage for genomic and transcriptomic *Pgt* datasets identified 46 *Pgt* datasets with low coverage that were excluded from further analysis. Analyses was conducted on 165 *Pgt* transcriptomics and genomic datasets that were independently aligned to the *Pgt* reference genome (CRL 75-36-700-3 [27]). *Pgt* datasets with coverage in over 15% of genes and allele frequencies at biallelic SNP sites displaying a normal distribution at 0.5 were retained for future analysis (labelled as ‘PASS’ in green). In contrast, 46 *Pgt* datasets with low coverage, lacking coverage for an average of 13,909 genes (S.D. ±2,344 genes) out of a total of 16,309 predicted genes in the *Pgt* reference genome (marked as ‘FAIL’ in red), as well as 15 *Pgt* datasets with allele frequencies deviating from the normal distribution at 0.5 (marked as ‘FAIL AF’ in purple) were excluded from further analysis.

**Additional file 4**: **Fig. S3**. Multivariate discriminant analysis of principal components (DAPC) analysis supports the clustering of 86 *Pgt* isolates in 12 genetic groups. (A) DAPC analysis was performed using 19,250 biallelic single polymorphism (SNP) sites across *k*-values from 3 to 15, clustering the 86 *Pgt* isolates into various genetic groups. (B) The Bayesian information criterion (BIC) indicated an initial optimal *k*-value at 5, marking the first inflection point and dividing the *Pgt* isolates into five main groups. (C) A second round of DAPC analysis was conducted on groups with more than 10 *Pgt* individuals, further resolving within-group variation. This secondary analysis indicated optimal *k*-values of 3 for the first two groups and 4 for the third, ultimately resulting in 12 distinct genetic groups.

**Additional file 5**: **Fig. S4**. A subset of 392 *Pgt* genes with > 0.13 SNPs/bp was sufficient to accurately reconstruct the phylogenetic topology, even with 40% of genes excluded. Randomly selected 60% subsets of the 392 highly polymorphic *Pgt* genes with > 0.13 SNPs/bp were used to generate cladograms of the 86 *Pgt* isolates using an approximate maximum-likelihood model.

**Additional file 6**: **Fig. S5**. Gene Ontology (GO) annotations for the 276 highly polymorphic *Pgt* genes showed that, among the 89 genes with annotated protein functions, half are associated with catalytic activities (GO:0003824). A total of 70, 54 and 56 *Pgt* genes encoded proteins annotated with molecular functions, associated with cellular components and/or are involved in biological processes, respectively.

**Additional file 7**: **Fig. S6**. The MultiQC report from analysis of 12 *Pgt*-infected field samples collected in Kenya and Ethiopia shows that sequence data was successfully generated for an average of 78% of the 276 polymorphic *Pgt* genes. Pools A-D, individual primer pools; % Dups, percentage of duplicated reads.

**Additional file 8**: **Fig. S7**. Primers successfully amplified the genes *Cyp51*, *SdhA*, *SdhB*, *SdhC* and *SdhD* in *Pgt* isolates UK-01, UK-03 and UK-13. PCR amplification of the five genes was confirmed following agarose gel electrophoresis, with a no template control (NTC) containing water included for each primer pair. bp, base pairs.

**Additional file 9**: **Fig. S8**. Sequence alignments of five fungicide target genes * – Cyp51*, *SdhA*, *SdhB*, *SdhC* and *SdhD–* across five *Pgt* isolates with available reference genomes. Primers were designed from conserved regions within the orthologous sequences for *Cyp51*, *SdhA*, *SdhB*, *SdhC* and *SdhD* extracted from the *Pgt* reference genomes (CRL 75-36-700-3 [27], 99KS76A [44], 21 − 0, Ug99 [21], UK-01 [23]).

**Additional file 10**: **Dataset S1**. Phylogenetic analysis of the 86 *Pgt* isolates and 2 *Pga* isolates used to assign *Pgt*-infected field samples to different genetic groups. Newick format.

**Additional file 11**: **Dataset S2**. Phylogenetic analysis of the 86 *Pgt* isolates, for genes with an average > 0.13 SNP/bp. Newick format.

**Additional file 12**: **Dataset S3**. Phylogenetic analysis of *Pgt*-infected wheat field samples from Kenya and Ethiopia, along with the 102 global *Pgt* and 2 outgroup *Puccinia graminis f. sp. avenae* (*Pga*) isolates used in MARPLE diagnostics. Newick format.

## Data Availability

All custom computer code and an easy-to-follow guide for installing prerequisite software has been deposited on GitHub (https://github.com/SaundersLab/marple.git). An interactive phylogenetic tree of the isolates included in MARPLE is available on Nextstrain (https://nextstrain.org/community/saunderslab/WheatRust/PGT-marple). Phylogenies are also provided for detailed interrogation in Newick format (Additional files 10-12). The nanopore data that supports the findings of this study has been deposited in the Sequence Read Archive (BioProject: PRJNA1214760).

## References

[1] Stukenbrock E, Gurr S. Address the growing urgency of fungal disease in crops. Nature. 2023;617(7959):31–4. 37130937 10.1038/d41586-023-01465-4

[2] Hawkins NJ, Fraaije BA. Contrasting levels of genetic predictability in the evolution of resistance to major classes of fungicides. Mol Ecol. 2021;30(21):5318–27. 33706414 10.1111/mec.15877

[3] Hariharan G, Prasannath K. Recent advances in molecular diagnostics of fungal plant pathogens: A Mini review. Front Cell Infect Microbiol. 2020;10:600234. 33505921 10.3389/fcimb.2020.600234 PMC7829251

[4] Cumagun CJ. Plant pathology: BoD–Books on Demand; 2012.

[5] Donoso A, Valenzuela S. In-field molecular diagnosis of plant pathogens: recent trends and future perspectives. Plant Pathol. 2018;67(7):1451–61.

[6] Kanj S. Rapid diagnosis of fungal infections: Impact on stewardship. In: 9th Trends in Medical Mycology Conference 2019: 2019; Nice, France: The Fungal Infection Trust; 2019.

[7] Hof H. Critical annotations to the use of Azole antifungals for plant protection. Antimicrob Agents Chemother. 2001;45(11):2987–90. 11600346 10.1128/AAC.45.11.2987-2990.2001 PMC90772

[8] Steffens JJ, Pell EJ, Tien M. Mechanisms of fungicide resistance in phytopathogenic fungi. Curr Opin Biotechnol. 1996;7(3):348–55. 8785443 10.1016/s0958-1669(96)80043-7

[9] Pintye A, Bacsó R, Kovács GM. Trans-kingdom fungal pathogens infecting both plants and humans, and the problem of Azole fungicide resistance. Front Microbiol 2024, 15. 10.3389/fmicb.2024.1354757 PMC10896089 38410389

[10] Li S, Li X, Zhang H, Wang Z, Xu H. The research progress in and perspective of potential fungicides: succinate dehydrogenase inhibitors. Bioorg Med Chem. 2021;50:116476. 34757244 10.1016/j.bmc.2021.116476

[11] Tsang CC, Teng JLL, Lau SKP, Woo PCY. Rapid genomic diagnosis of fungal infections in the age of Next-Generation sequencing. J Fungi (Basel) 2021, 7(8). 10.3390/jof7080636 PMC8398552 34436175

[12] Panth M, Hassler SC, Baysal-Gurel F. Methods for management of soilborne diseases in crop production. Agriculture-Basel 2020, 10(1).

[13] Mikheyev AS, Tin MMY. A first look at the Oxford nanopore minion sequencer. Mol Ecol Resour. 2014;14(6):1097–102. 25187008 10.1111/1755-0998.12324

[14] Quick J, Loman NJ, Duraffour S, Simpson JT, Severi E, Cowley L, Bore JA, Koundouno R, Dudas G, Mikhail A, et al. Real-time, portable genome sequencing for Ebola surveillance. Nature. 2016;530(7589):228–32. 26840485 10.1038/nature16996 PMC4817224

[15] Faria NR, Sabino EC, Nunes MR, Alcantara LC, Loman NJ, Pybus OG. Mobile real-time surveillance of Zika virus in Brazil. Genome Med. 2016;8(1):97. 27683027 10.1186/s13073-016-0356-2 PMC5041528

[16] Boykin L, Ghalab A, Rossitto De Marchi B, Savill A, Wainaina M, Kinene J, Lamb T, Rodrigues S, Kehoe M, Ndunguru M et al. J: Real time portable genome sequencing for global food security. F1000Research 2018;7(1101).

[17] Saunders DGO, Hodson D. Answering the call for real-time plant disease diagnostics in resource-poor regions. In: On Biology. BMC 2019.

[18] Fei W, Liu Y. Biotrophic fungal pathogens: a critical overview. Appl Biochem Biotechnol. 2023;195(1):1–16. 35951248 10.1007/s12010-022-04087-0

[19] Radhakrishnan GV, Cook NM, Bueno-Sancho V, Lewis CM, Persoons A, Mitiku AD, Heaton M, Davey PE, Abeyo B, Alemayehu Y, et al. MARPLE, a point-of-care, strain-level disease diagnostics and surveillance tool for complex fungal pathogens. BMC Biol. 2019;17(1):65. 31405370 10.1186/s12915-019-0684-y PMC6691556

[20] Leonard KJ, Szabo LJ. Stem rust of small grains and grasses caused by Puccinia graminis. Mol Plant Pathol. 2005;6(2):99–111. 20565642 10.1111/j.1364-3703.2005.00273.x

[21] Li F, Upadhyaya NM, Sperschneider J, Matny O, Hoa NP, Mago R, Raley C, Miller ME, Silverstein KAT, Henningsen E et al. Emergence of the Ug99 lineage of the wheat stem rust pathogen through somatic hybridisation. Nat Commun 2019, 10. 10.1038/s41467-019-12927-7 PMC6838127 31699975

[22] Guo Y, Betzen B, Salcedo A, He F, Bowden RL, Fellers JP, Jordan KW, Akhunova A, Rouse MN, Szabo LJ, et al. Population genomics of *Puccinia graminis* F.sp. *Tritici* highlights the role of admixture in the origin of virulent wheat rust races. Nat Commun. 2022;13(1):6287. 36271077 10.1038/s41467-022-34050-w PMC9587050

[23] Lewis CM, Persoons A, Bebber DP, Kigathi RN, Maintz J, Findlay K, Bueno-Sancho V, Corredor-Moreno P, Harrington SA, Kangara N, et al. Potential for re-emergence of wheat stem rust in the united Kingdom. Commun Biol. 2018;1:13. 30271900 10.1038/s42003-018-0013-y PMC6053080

[24] Chen J, Upadhyaya NM, Ortiz D, Sperschneider J, Li F, Bouton C, Breen S, Dong C, Xu B, Zhang X, et al. Loss of *AvrSr50* by somatic exchange in stem rust leads to virulence for *Sr50* resistance in wheat. Science. 2017;358(6370):1607–10. 29269475 10.1126/science.aao4810

[25] Rutter WB, Salcedo A, Akhunova A, He F, Wang SC, Liang HQ, Bowden RL, Akhunov E. Divergent and convergent modes of interaction between wheat and *Puccinia graminis* F. sp *tritici* isolates revealed by the comparative gene co-expression network and genome analyses. BMC Genomics 2017, 18. 10.1186/s12864-017-3678-6 PMC5389088 28403814

[26] Sperschneider J, Jones AW, Nasim J, Xu B, Jacques S, Zhong CC, Upadhyaya NM, Mago R, Hu YH, Figueroa M et al. The stem rust F.ngus *Puccinia graminis* F. Sp. *tritici* induces centromeric small RNAs during late infection that are associated with genome-wide DNA methylation. BMC Biol 2021, 19(1). 10.1186/s12915-021-01123-z PMC8444563 34526021

[27] Duplessis S, Cuomo CA, Lin YC, Aerts A, Tisserant E, Veneault-Fourrey C, Joly DL, Hacquard S, Amselem J, Cantarel BL, et al. Obligate biotrophy features unraveled by the genomic analysis of rust fungi. Proc Natl Acad Sci U S A. 2011;108(22):9166–71. 21536894 10.1073/pnas.1019315108 PMC3107277

[28] Li H, Durbin R. Fast and accurate short read alignment with Burrows-Wheeler transform. Bioinformatics. 2009;25(14):1754–60. 19451168 10.1093/bioinformatics/btp324 PMC2705234

[29] Dobin A, Davis CA, Schlesinger F, Drenkow J, Zaleski C, Jha S, Batut P, Chaisson M, Gingeras TR. STAR: ultrafast universal RNA-seq aligner. Bioinformatics. 2013;29(1):15–21. 23104886 10.1093/bioinformatics/bts635 PMC3530905

[30] McKenna A, Hanna M, Banks E, Sivachenko A, Cibulskis K, Kernytsky A, Garimella K, Altshuler D, Gabriel S, Daly M, et al. The genome analysis toolkit: a mapreduce framework for analyzing next-generation DNA sequencing data. Genome Res. 2010;20(9):1297–303. 20644199 10.1101/gr.107524.110 PMC2928508

[31] Hubbard A, Lewis CM, Yoshida K, Ramirez-Gonzalez RH, de Vallavieille-Pope C, Thomas J, Kamoun S, Bayles R, Uauy C, Saunders DG. Field pathogenomics reveals the emergence of a diverse wheat yellow rust population. Genome Biol. 2015;16(1):23. 25723868 10.1186/s13059-015-0590-8 PMC4342793

[32] Savva L, Saunders DGO. *Pgt* MARPLE diagnostics. In: https://github.com/SaundersLab/marple-pgt-paper. GitHub; 2024.

[33] Price MN, Dehal PS, Arkin AP. FastTree 2–approximately maximum-likelihood trees for large alignments. PLoS ONE. 2010;5(3):e9490. 20224823 10.1371/journal.pone.0009490 PMC2835736

[34] Letunic I, Bork P. Interactive tree of life (iTOL) v6: recent updates to the phylogenetic tree display and annotation tool. Nucleic Acids Res. 2024;52(W1):W78–82. 38613393 10.1093/nar/gkae268 PMC11223838

[35] Jombart T, Ahmed I. Adegenet 1.3-1: new tools for the analysis of genome-wide SNP data. Bioinformatics. 2011;27(21):3070–1. 21926124 10.1093/bioinformatics/btr521 PMC3198581

[36] Savva L, Saunders DGO. MARPLE diagnostics. In: https://githubcom/SaundersLab/marple. GitHub; 2024.

[37] Schwessinger B, Sperschneider J, Cuddy WS, Garnica DP, Miller ME, Taylor JM, Dodds PN, Figueroa M, Park RF, Rathjen JP. A Near-Complete Haplotype-Phased Genome of the Dikaryotic Wheat Stripe Rust Fungus *Puccinia striiformis* f. sp. *tritici* Reveals High Interhaplotype Diversity. *mBio* 2018, 9(1). 10.1128/mBio.02275-17 PMC5821087 29463659

[38] Li H, Handsaker B, Wysoker A, Fennell T, Ruan J, Homer N, Marth G, Abecasis G, Durbin R. Genome project data processing S: the sequence alignment/map format and samtools. Bioinformatics. 2009;25(16):2078–9. 19505943 10.1093/bioinformatics/btp352 PMC2723002

[39] Stamatakis A. RAxML version 8: a tool for phylogenetic analysis and post-analysis of large phylogenies. Bioinformatics. 2014;30(9):1312–3. 24451623 10.1093/bioinformatics/btu033 PMC3998144

[40] Borozan L, Matijevic D, Canzar S. Properties of the generalized Robinson-Foulds metric. *2019 42nd International Convention on Information and Communication Technology, Electronics and Microelectronics (Mipro)* 2019:330–335.

[41] Bogdanowicz D, Giaro K, Wróbel B. TreeCmp: comparison of trees in polynomial time. Evol Bioinform. 2012;8:475–87.

[42] Untergasser A, Cutcutache I, Koressaar T, Ye J, Faircloth BC, Remm M, Rozen SG. Primer3–new capabilities and interfaces. Nucleic Acids Res. 2012;40(15):e115. 22730293 10.1093/nar/gks596 PMC3424584

[43] Zhu T, Wang L, Rimbert H, Rodriguez JC, Deal KR, De Oliveira R, Choulet F, Keeble-Gagnère G, Tibbits J, Rogers J, et al. Optical maps refine the bread wheat *Triticum aestivum cv.* Chinese Spring genome assembly. Plant J. 2021;107(1):303–14. 33893684 10.1111/tpj.15289 PMC8360199

[44] Salcedo A, Rutter W, Wang S, Akhunova A, Bolus S, Chao S, Anderson N, De Soto MF, Rouse M, Szabo L, et al. Variation in the *AvrSr35* gene determines *Sr35* resistance against wheat stem rust race Ug99. Science. 2017;358(6370):1604–6. 29269474 10.1126/science.aao7294 PMC6518949

[45] Brown SS, Chen YW, Wang M, Clipson A, Ochoa E, Du MQ. PrimerPooler: automated primer pooling to prepare library for targeted sequencing. Biol Methods Protoc. 2017;2(1):bpx006. 32161789 10.1093/biomethods/bpx006 PMC6994079

[46] Klopfenstein DV, Zhang L, Pedersen BS, Ramirez F, Warwick Vesztrocy A, Naldi A, Mungall CJ, Yunes JM, Botvinnik O, Weigel M, et al. GOATOOLS: A Python library for gene ontology analyses. Sci Rep. 2018;8(1):10872. 30022098 10.1038/s41598-018-28948-z PMC6052049

[47] Bendtsen JD, Nielsen H, von Heijne G, Brunak S. Improved prediction of signal peptides: signalp 3.0. J Mol Biol. 2004;340(4):783–95. 15223320 10.1016/j.jmb.2004.05.028

[48] Sperschneider J, Dodds PN. EffectorP 3.0: prediction of apoplastic and cytoplasmic effectors in Fungi and oomycetes. Mol Plant Microbe Interact. 2022;35(2):146–56. 34698534 10.1094/MPMI-08-21-0201-R

[49] Molder F, Jablonski KP, Letcher B, Hall MB, Tomkins-Tinch CH, Sochat V, Forster J, Lee S, Twardziok SO, Kanitz A et al. Sustainable data analysis with Snakemake. *F1000Res* 2021, 10:33. 10.12688/f1000research.29032.1 PMC8114187 34035898

[50] Steinig E, Coin L. Nanoq: ultra-fast quality control for nanopore reads. J Open Source Softw. 2022;7(69):2991.

[51] Pertea G, Pertea M. GFF Utilities: GffRead and GffCompare. *F1000Res* 2020, 9. 10.12688/f1000research.23297.1 PMC7222033 32489650

[52] Ewels P, Magnusson M, Lundin S, Kaller M. MultiQC: summarize analysis results for multiple tools and samples in a single report. Bioinformatics. 2016;32(19):3047–8. 27312411 10.1093/bioinformatics/btw354 PMC5039924

[53] Lewis CM, Morier-Gxoyiya C, Hubbard A, Nellist CF, Bebber DP, Saunders DGO. Resurgence of wheat stem rust infections in Western Europe: causes and how to curtail them. New Phytol. 2024;243(2):537–42. 38803104 10.1111/nph.19864

[54] Cook NM, Chng S, Woodman TL, Warren R, Oliver RP, Saunders DG. High F.equency of F.ngicide resistance-associated mutations in the wheat yellow rust pathogen *Puccinia striiformis* F. Sp. *tritici*. Pest Manag Sci. 2021;77(7):3358–71. 33786966 10.1002/ps.6380

[55] Larkin MA, Blackshields G, Brown NP, Chenna R, McGettigan PA, McWilliam H, Valentin F, Wallace IM, Wilm A, Lopez R, et al. Clustal W and clustal X version 2.0. Bioinformatics. 2007;23(21):2947–8. 17846036 10.1093/bioinformatics/btm404

[56] Tsushima A, Lewis CM, Flath K, Kildea S, Saunders DGO. Wheat stem rust recorded for the first time in decades in Ireland. Plant Pathol. 2022;71(4):890–900. 35873178 10.1111/ppa.13532 PMC9303354

[57] Seong K, Krasileva KV. Prediction of effector protein structures from fungal phytopathogens enables evolutionary analyses. Nat Microbiol. 2023;8(1):174–. 36604508 10.1038/s41564-022-01287-6 PMC9816061

[58] Stammler G, Cordero J, Koch A, Semar M, Schlehuber S. Role of the Y134F mutation in *cyp51* and overexpression of *cyp51* in the sensitivity response of *Puccinia triticina* to Epoxiconazole. Crop Prot. 2009;28(10):891–7.

[59] Fisher MC, Henk DA, Briggs CJ, Brownstein JS, Madoff LC, McCraw SL, Gurr SJ. Emerging fungal threats to animal, plant and ecosystem health. Nature. 2012;484(7393):186–94. 22498624 10.1038/nature10947 PMC3821985

[60] Olivera P, Newcomb M, Szabo LJ, Rouse M, Johnson J, Gale S, Luster DG, Hodson D, Cox JA, Burgin L, et al. Phenotypic and genotypic characterization of race TKTTF of *Puccinia graminis* F. sp *tritici* that caused a wheat stem rust epidemic in Southern Ethiopia in 2013-14. Phytopathology. 2015;105(7):917–28. 25775107 10.1094/PHYTO-11-14-0302-FI

[61] Saunders DGO, Pretorius ZA, Hovmoller MS. Tackling the re-emergence of wheat stem rust in Western Europe. Commun Biol. 2019;2:51. 30729187 10.1038/s42003-019-0294-9 PMC6361993

[62] Hodson D. Successful surveillance results in early first detection of Ug99 in South Asia. Volume 2024. CIMMYT; 2024.

[63] Rafiei V, Velez H, Tzelepis G. The role of glycoside hydrolases in phytopathogenic Fungi and oomycetes virulence. Int J Mol Sci 2021, 22(17). 10.3390/ijms22179359 PMC8431085 34502268

[64] Krishnan P, Ma X, McDonald BA, Brunner PC. Widespread signatures of selection for secreted peptidases in a fungal plant pathogen. BMC Evol Biol. 2018;18(1):7. 29368587 10.1186/s12862-018-1123-3 PMC5784588

[65] Yin Y, Miao J, Shao W, Liu X, Zhao Y, Ma Z. Fungicide resistance: progress in Understanding mechanism, monitoring, and management. Phytopathology. 2023;113(4):707–18. 36624725 10.1094/PHYTO-10-22-0370-KD

[66] Jorgensen LN, Matzen N, Leitzke R, Thomas JE, O’Driscoll A, Klocke B, Maumene C, Lindell I, Wahlquist K, Zemeca L et al. Management of rust in wheat using IPM principles and alternative products. Agriculture-Basel 2024, 14(6).

[67] Koster J, Rahmann S. Snakemake-a scalable bioinformatics workflow engine. Bioinformatics. 2018;34(20):3600. 29788404 10.1093/bioinformatics/bty350

