# Supplementary figures and images for: A portable, nanopore-based genotyping platform for near real-time detection of Puccinia graminis f. sp. tritici lineages and fungicide sensitivity
Source: BMC Genomics. 2025 Apr 1;26:327. doi: 10.1186/s12864-025-11428-w (PMC11959956; doi:10.1186/s12864-025-11428-w)

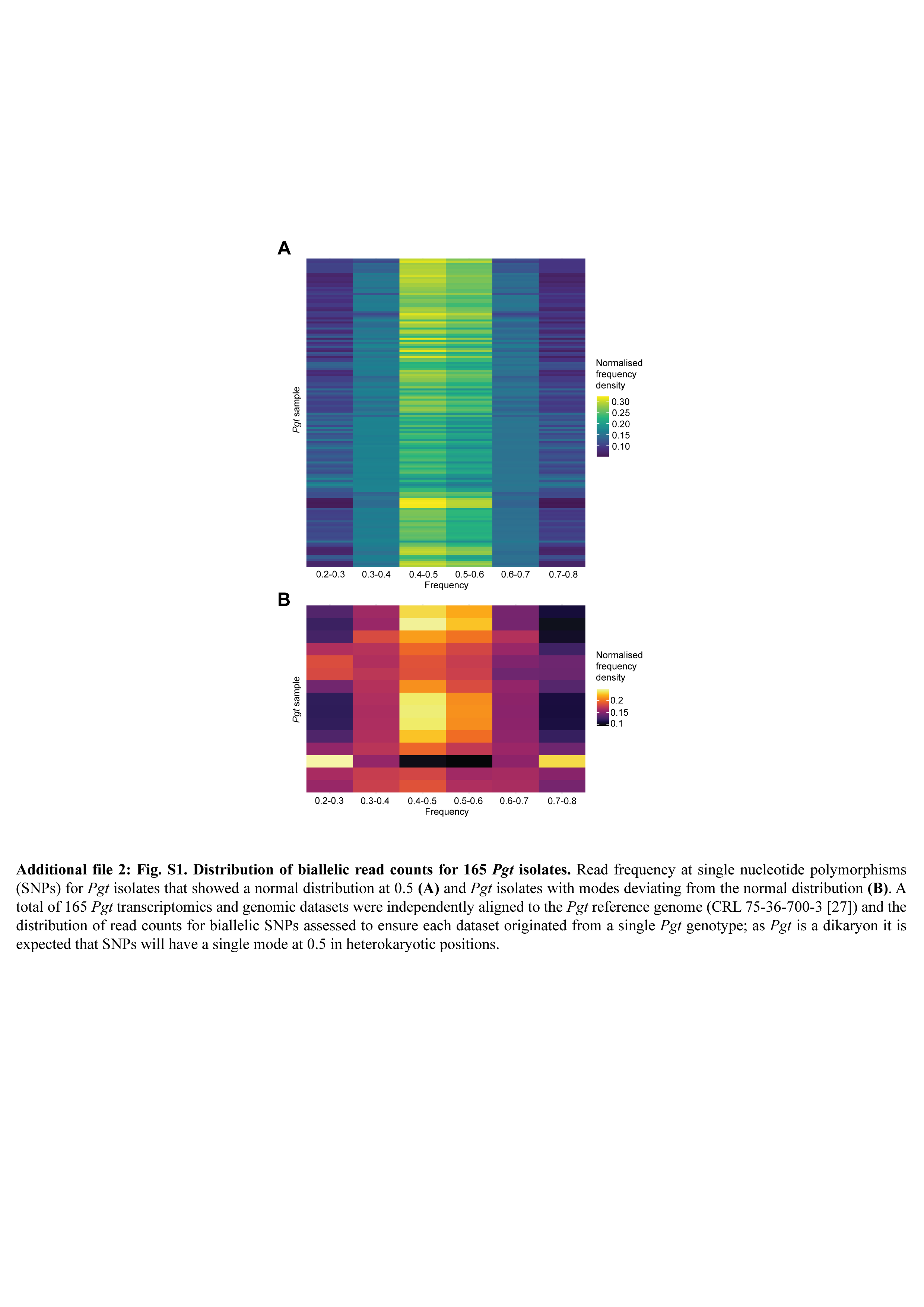

Supplement: Supplementary file 2 — Additional file 2: Fig. S1. Distribution of biallelic read counts for 165 Pgt isolates. Read frequency at single nucleotide polymorphisms (SNPs) for Pgt isolates that showed a normal distribution at 0.5 (A) and Pgt isolates with modes deviating from the normal distribution (B). A total of 165 Pgt transcriptomics and genomic datasets were independently aligned to the Pgt reference genome (CRL 75-36-700-3 [27]) and the distribution of read counts for biallelic SNPs assessed to ensure each dataset originated from a single Pgt genotype; as Pgt is a dikaryon it is expected that SNPs will have a single mode at 0.5 in heterokaryotic positions. [file 12864_2025_11428_MOESM2_ESM.tif]

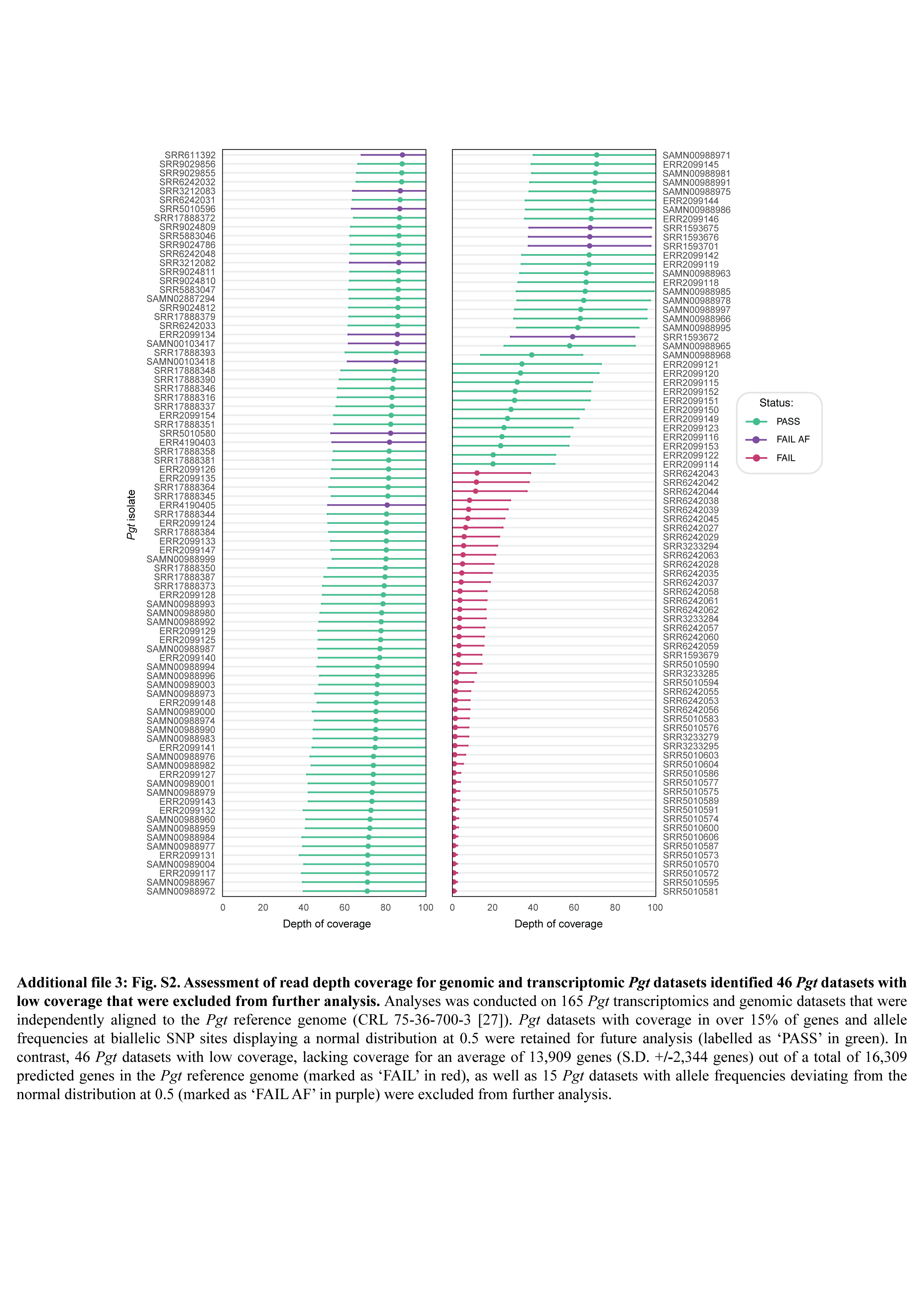

Supplement: Supplementary file 3 — Additional file 3: Fig. S2. Assessment of read depth coverage for genomic and transcriptomic Pgt datasets identified 46 Pgt datasets with low coverage that were excluded from further analysis. Analyses was conducted on 165 Pgt transcriptomics and genomic datasets that were independently aligned to the Pgt reference genome (CRL 75-36-700-3 [27]). Pgt datasets with coverage in over 15% of genes and allele frequencies at biallelic SNP sites displaying a normal distribution at 0.5 were retained for future analysis (labelled as ‘PASS’ in green). In contrast, 46 Pgt datasets with low coverage, lacking coverage for an average of 13,909 genes (S.D. ±2,344 genes) out of a total of 16,309 predicted genes in the Pgt reference genome (marked as ‘FAIL’ in red), as well as 15 Pgt datasets with allele frequencies deviating from the normal distribution at 0.5 (marked as ‘FAIL AF’ in purple) were excluded from further analysis. [file 12864_2025_11428_MOESM3_ESM.tif]

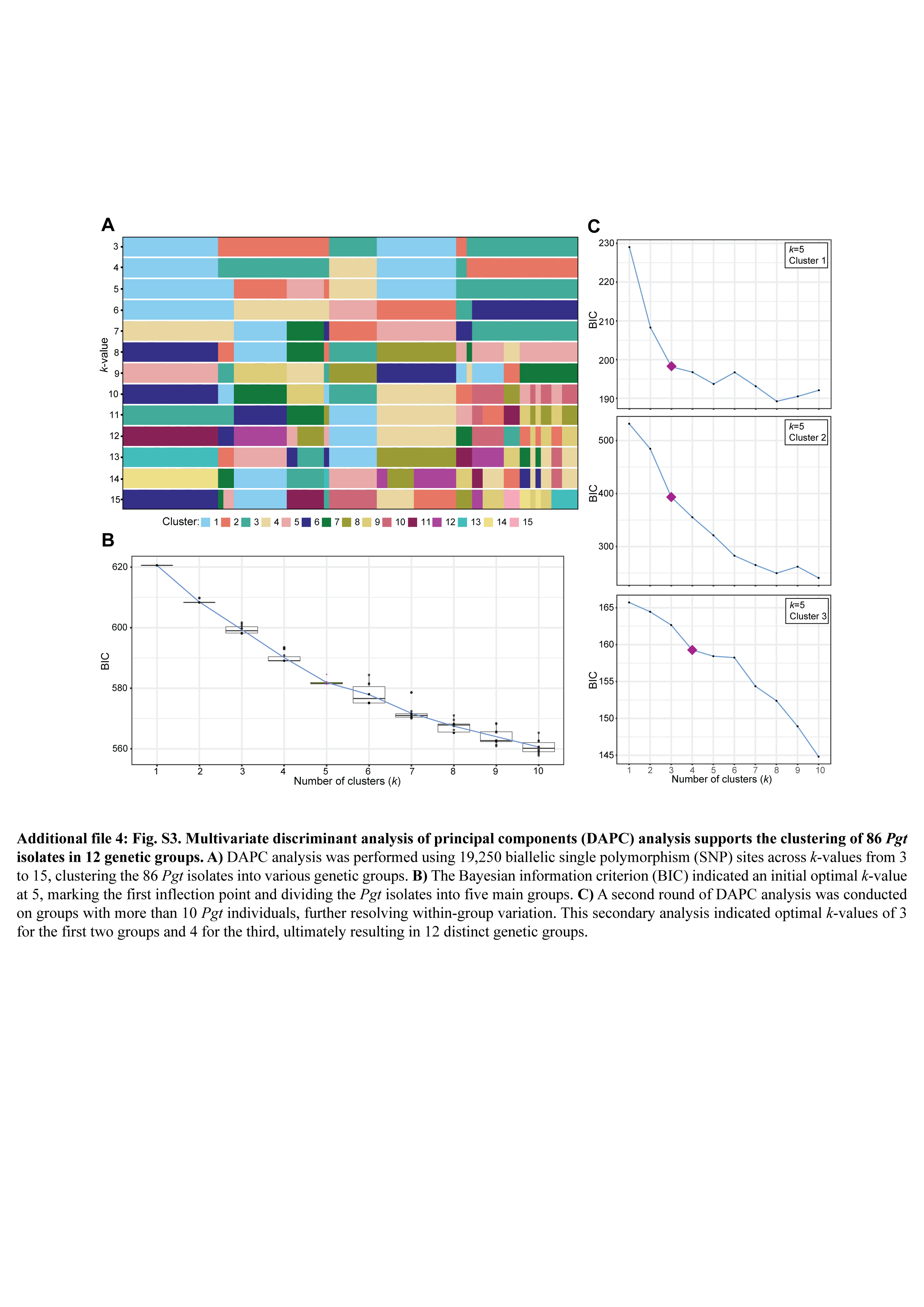

Supplement: Supplementary file 4 — Additional file 4: Fig. S3. Multivariate discriminant analysis of principal components (DAPC) analysis supports the clustering of 86 Pgt isolates in 12 genetic groups. (A) DAPC analysis was performed using 19,250 biallelic single polymorphism (SNP) sites across k-values from 3 to 15, clustering the 86 Pgt isolates into various genetic groups. (B) The Bayesian information criterion (BIC) indicated an initial optimal k-value at 5, marking the first inflection point and dividing the Pgt isolates into five main groups. (C) A second round of DAPC analysis was conducted on groups with more than 10 Pgt individuals, further resolving within-group variation. This secondary analysis indicated optimal k-values of 3 for the first two groups and 4 for the third, ultimately resulting in 12 distinct genetic groups. [file 12864_2025_11428_MOESM4_ESM.tif]

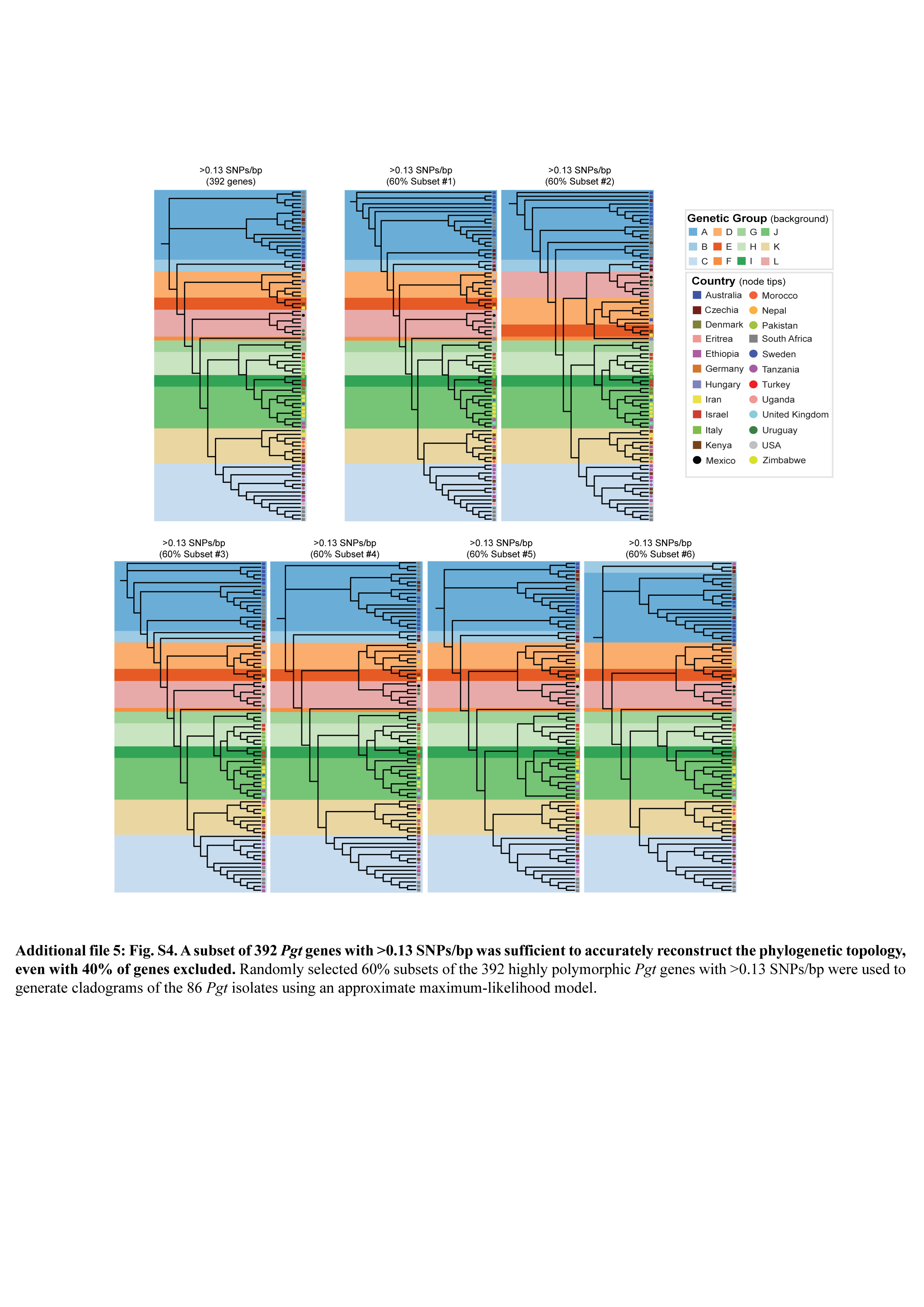

Supplement: Supplementary file 5 — Additional file 5: Fig. S4. A subset of 392 Pgt genes with > 0.13 SNPs/bp was sufficient to accurately reconstruct the phylogenetic topology, even with 40% of genes excluded. Randomly selected 60% subsets of the 392 highly polymorphic Pgt genes with > 0.13 SNPs/bp were used to generate cladograms of the 86 Pgt isolates using an approximate maximum-likelihood model. [file 12864_2025_11428_MOESM5_ESM.tif]

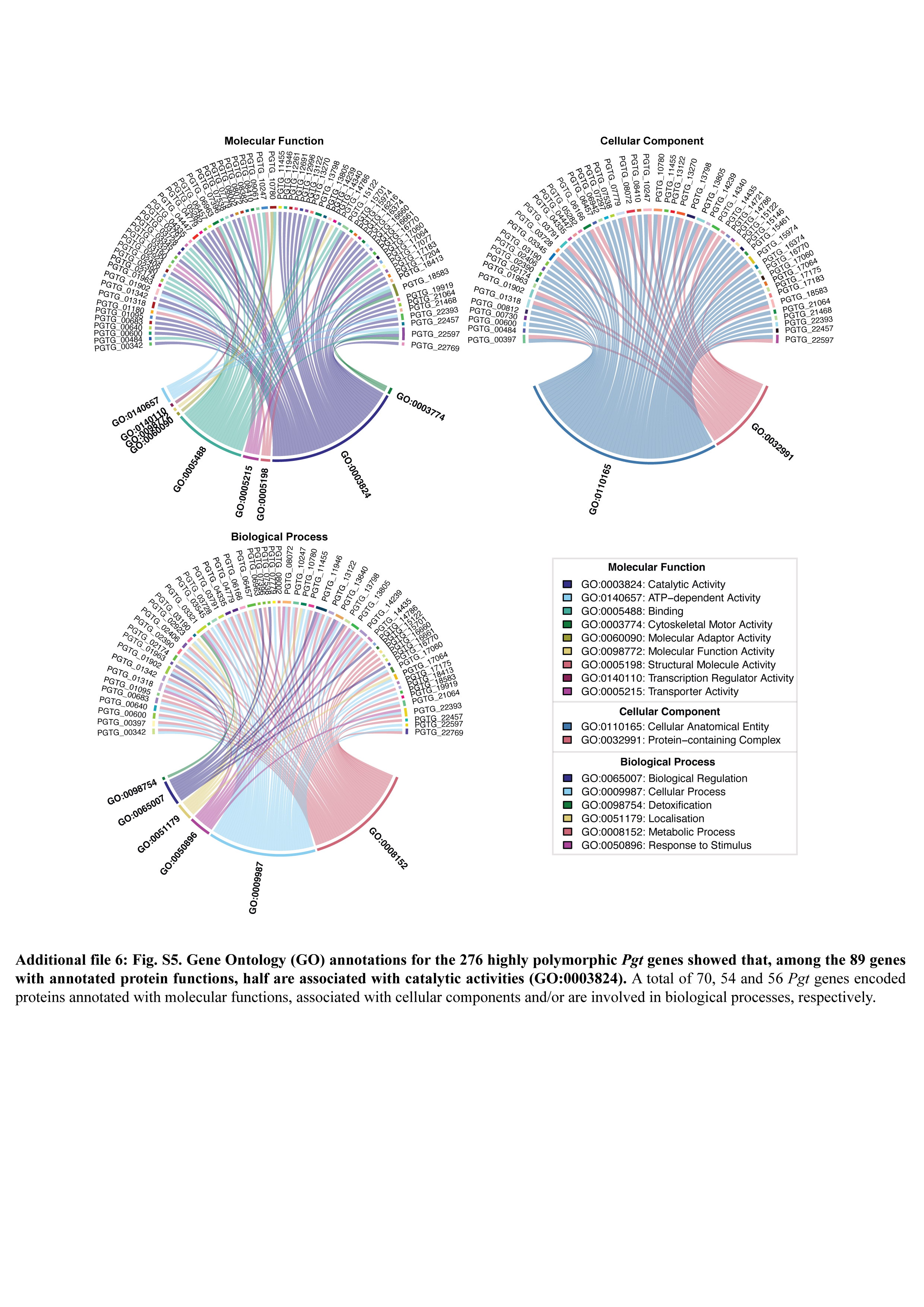

Supplement: Supplementary file 6 — Additional file 6: Fig. S5. Gene Ontology (GO) annotations for the 276 highly polymorphic Pgt genes showed that, among the 89 genes with annotated protein functions, half are associated with catalytic activities (GO:0003824). A total of 70, 54 and 56 Pgt genes encoded proteins annotated with molecular functions, associated with cellular components and/or are involved in biological processes, respectively. [file 12864_2025_11428_MOESM6_ESM.tif]

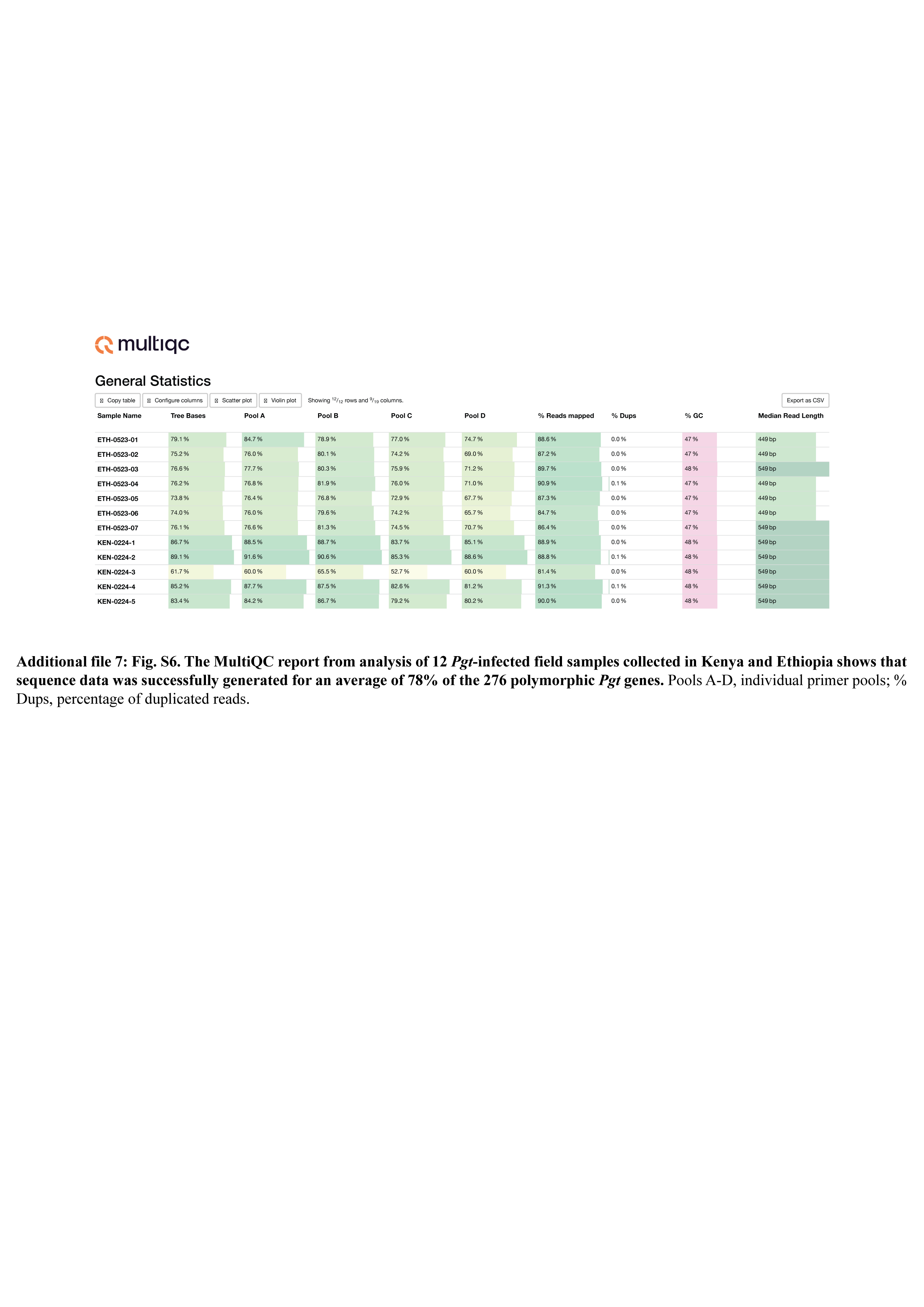

Supplement: Supplementary file 7 — Additional file 7: Fig. S6. The MultiQC report from analysis of 12 Pgt-infected field samples collected in Kenya and Ethiopia shows that sequence data was successfully generated for an average of 78% of the 276 polymorphic Pgt genes. Pools A-D, individual primer pools; % Dups, percentage of duplicated reads. [file 12864_2025_11428_MOESM7_ESM.tif]

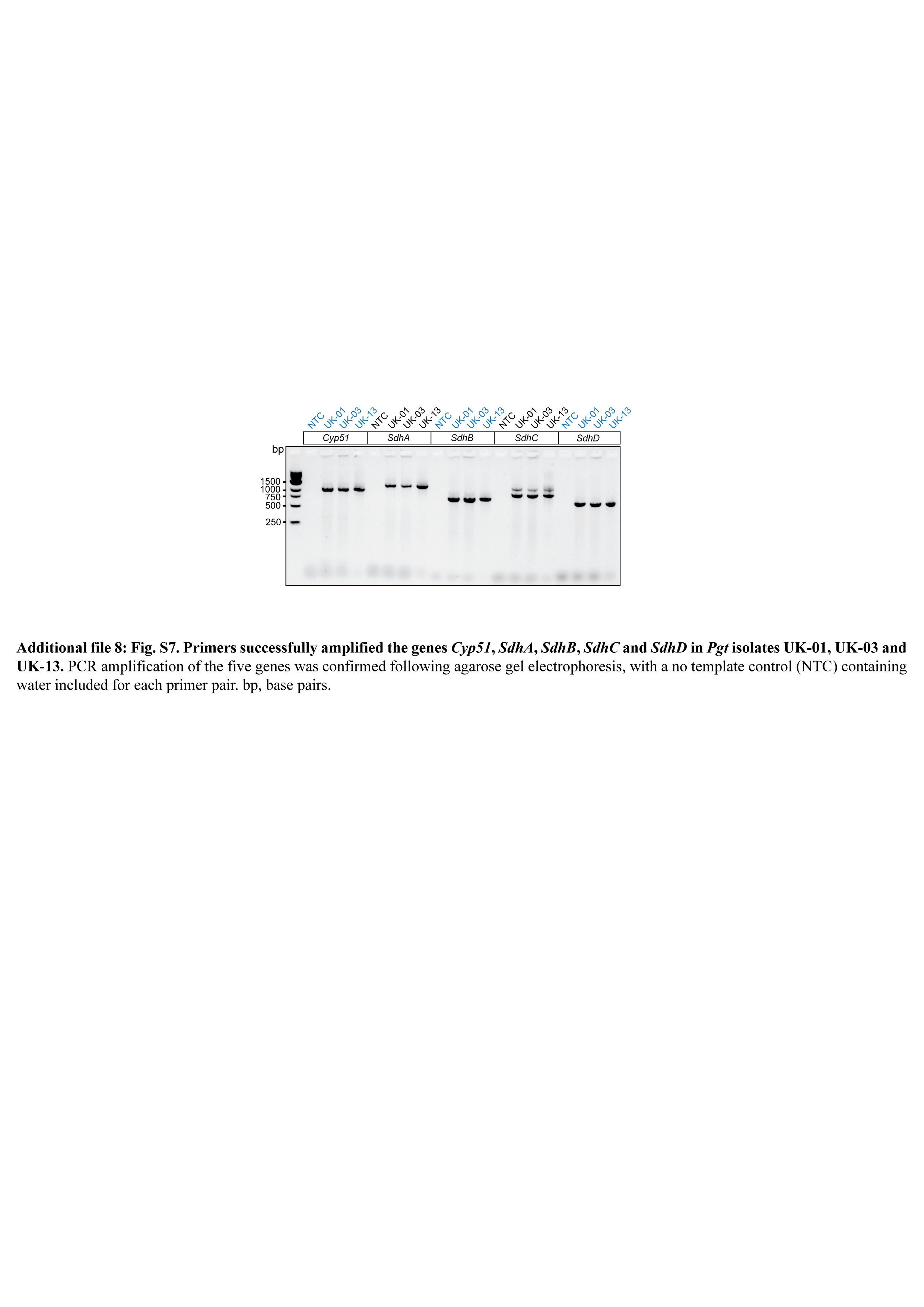

Supplement: Supplementary file 8 — Additional file 8: Fig. S7. Primers successfully amplified the genes Cyp51, SdhA, SdhB, SdhC and SdhD in Pgt isolates UK-01, UK-03 and UK-13. PCR amplification of the five genes was confirmed following agarose gel electrophoresis, with a no template control (NTC) containing water included for each primer pair. bp, base pairs. [file 12864_2025_11428_MOESM8_ESM.tif]

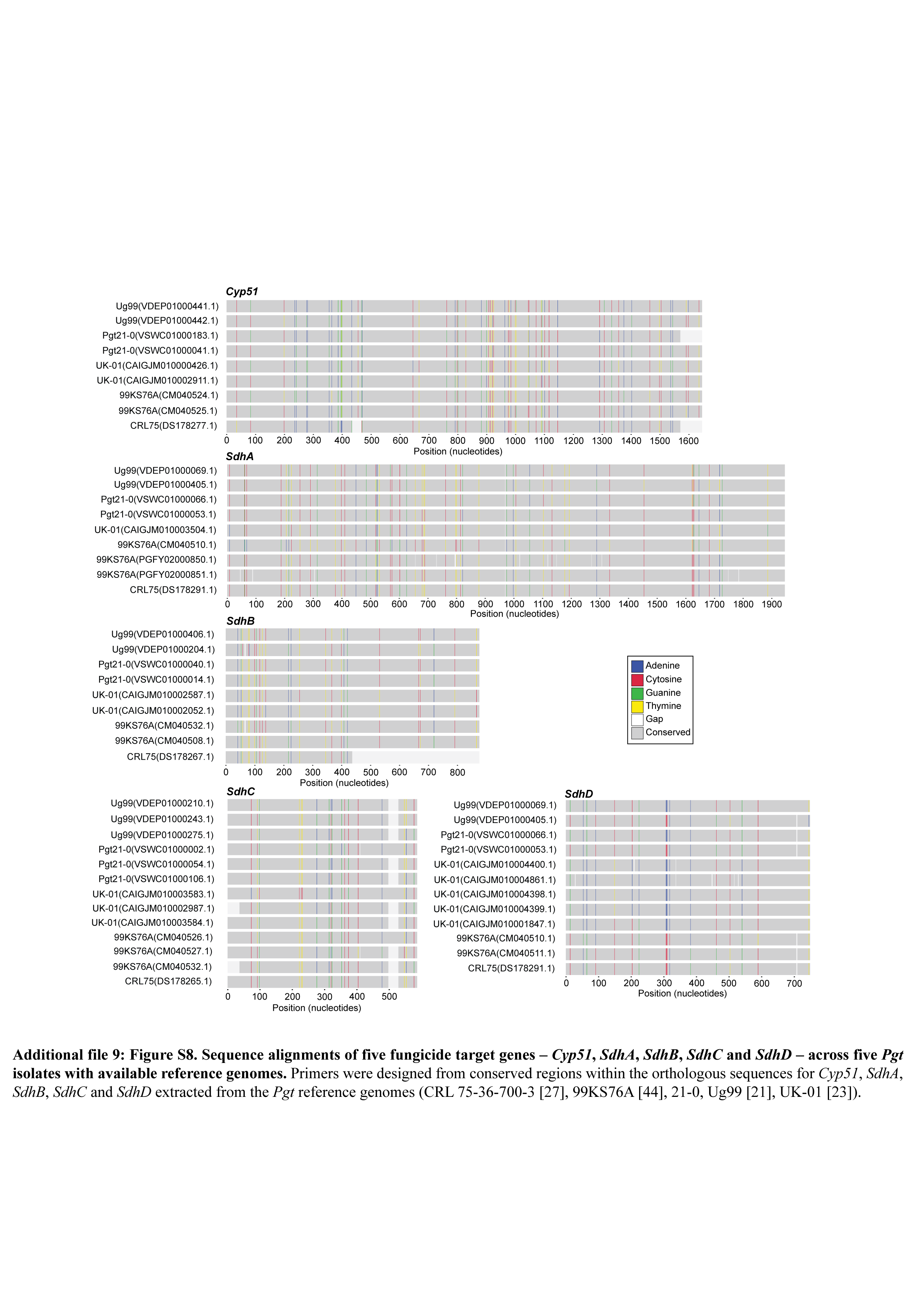

Supplement: Supplementary file 9 — Additional file 9: Fig. S8. Sequence alignments of five fungicide target genes – Cyp51, SdhA, SdhB, SdhC and SdhD– across five Pgt isolates with available reference genomes. Primers were designed from conserved regions within the orthologous sequences for Cyp51, SdhA, SdhB, SdhC and SdhD extracted from the Pgt reference genomes (CRL 75-36-700-3 [27], 99KS76A [44], 21 − 0, Ug99 [21], UK-01 [23]). [file 12864_2025_11428_MOESM9_ESM.tif]
